# Supplementary material for: Neutral evolution of snoRNA Host Gene long non-coding RNA affects cell fate control
Source: EMBO J. 2024 Jul 25;43(18):4049–67. doi: 10.1038/s44318-024-00172-8 (PMC11405852; doi:10.1038/s44318-024-00172-8)
Supplement: Supplementary file 1 — Appendix [file 44318_2024_172_MOESM1_ESM.pdf]

Appendix File

Appendix Figure S1. SNHGs in the genomic landscape. 2

Appendix Figure S2. SNHGs expression across human tissues. 4

Appendix Figure S3. Regulation and expression of epidermal SNHG in skin scRNA-seq datasets. 5

Appendix Figure S4. Epidermal SNHGs expression is controlled by multiple signalling pathways. 7

Appendix Figure S5. SNHG knockdown efficiency. 9

Appendix Figure S6. SNHG7 lncRNA affects epidermal stem cell proliferation and differentiation. 10

Appendix Figure S7. SNHG7 knockdown and overexpression efficiency in *A. trivirgatus* and *M. musculus* cells. 12

Appendix Figure S8. SNHG7 lncRNA affects the expression of targets of interacting miRNAs. 13

Appendix Figure S9. Evolution of SNHG-MRE interactions. 15

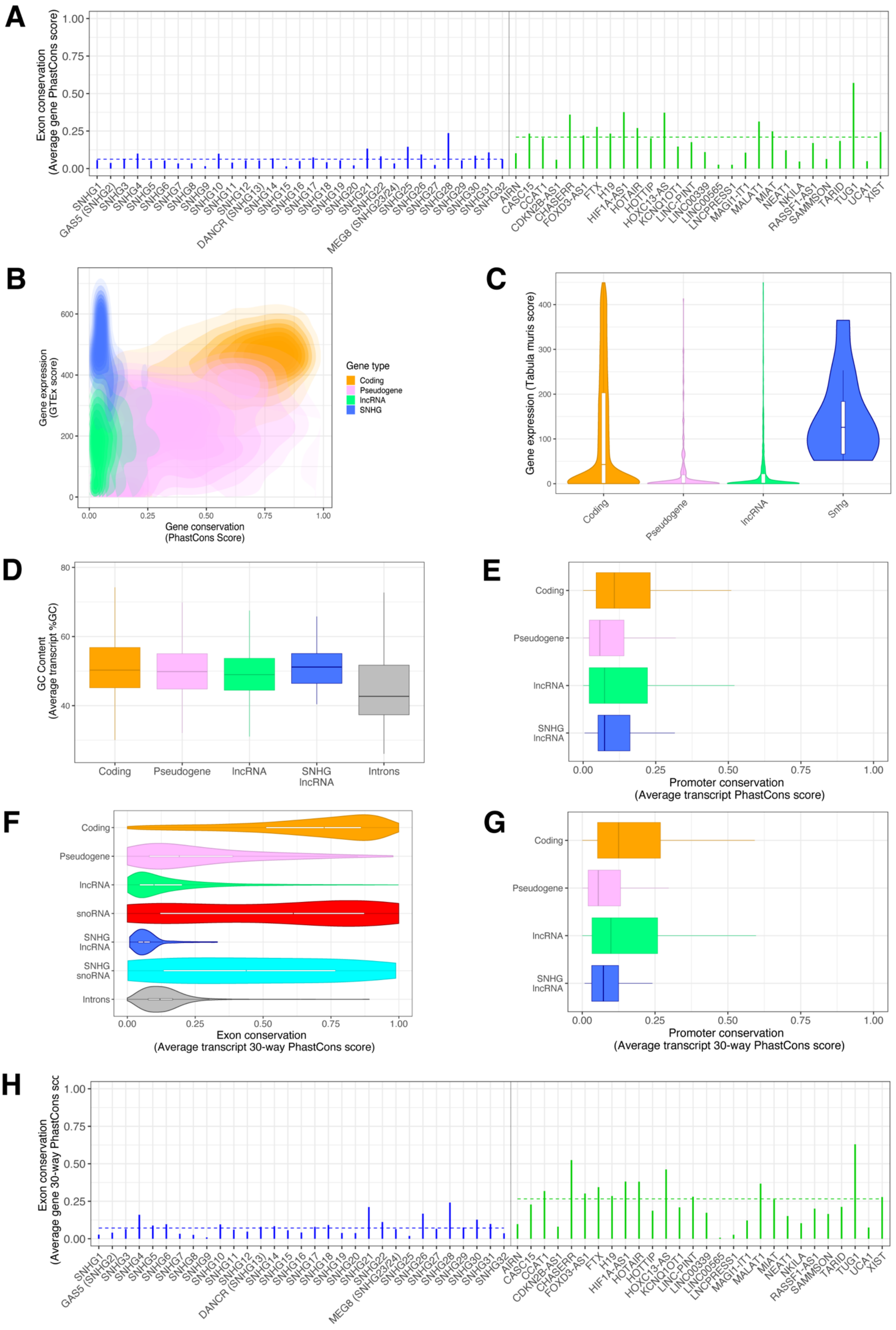
A
Exon conservation
(Average gene PhastCons score)
1.00
0.75
0.50
0.25
0.00
SNHG1
GAS5 (SNHG2)
SNHG3
SNHG4
SNHG5
SNHG6
SNHG7
SNHG8
SNHG9
SNHG10
SNHG11
SNHG12
DANCR (SNHG13)
SNHG14
SNHG15
SNHG16
SNHG17
SNHG18
SNHG19
SNHG20
SNHG21
MEG8 (SNHG23/24)
SNHG22
SNHG25
SNHG26
SNHG27
SNHG28
SNHG29
SNHG30
SNHG31
SNHG32
AIRN
CASC15
CCAT1
CDKN2B-AS1
CHASERR
FOXD3-AS1
FTX
H19
HIF1A-AS1
HOTAIR
HOTTIP
HOXC13-AS
KCNQ1OT1
LINC-PINT
LINC00339
LINC00565
LNCPRESS1
MAGI1-IT1
MALAT1
MIAT
NEAT1
NKILA
RASSF1-AS1
SAMMSON
TARID
TUG1
UCA1
XIST
B
Gene expression
(GTEx score)
Gene conservation
(PhastCons Score)
Gene type
Coding
Pseudogene
lncRNA
SNHG
C
Gene expression (Tabula muris score)
Coding
Pseudogene
lncRNA
Snhg
D
GC Content
(Average transcript %GC)
Coding
Pseudogene
lncRNA
SNHG
lncRNA
Introns
E
Coding
Pseudogene
lncRNA
SNHG
lncRNA
Promoter conservation
(Average transcript PhastCons score)
F
Coding
Pseudogene
lncRNA
snoRNA
SNHG
lncRNA
SNHG
snoRNA
Introns
Exon conservation
(Average transcript 30-way PhastCons score)
G
Coding
Pseudogene
lncRNA
SNHG
lncRNA
Promoter conservation
(Average transcript 30-way PhastCons score)
H
Exon conservation
(Average gene 30-way PhastCons sco

Appendix Figure S1. SNHGs in the genomic landscape. (**A**) Exonic conservation of SNHGs (blue) compared to a set of lncRNA with characterised functions (green). Dashed lines represent the mean conservation of each group. (**B**) Conservation and expression distribution of different transcript classes genome wide. (**C**) Gene expression scores of Snhg lncRNA compared to other classes of transcripts genome-wide in mouse. Boxplots within the violins indicate the median and the interquartile range. (**D**) Distribution of GC content in SNHG lncRNA compared to other classes of transcripts genome-wide. (**E**) Conservation of promoters in SNHGs and other gene classes. (**F**) Conservation of different classes of transcripts genome-wide evaluated by using PhastCons scores generated from alignment of 30 mammalian genomes (28 primates). (**G**) Conservation of promoters in SNHGs and other gene classes evaluated by using PhastCons scores generated from alignment of 30 mammalian genomes (28 primates). (**H**) Exonic conservation of SNHGs (blue) compared to a set of lncRNA with characterised functions (green) evaluated by using PhastCons scores generated from alignment of 30 mammalian genomes (28 primates). Dashed lines represent the mean conservation of each group. All boxplots indicate the median and the interquartile range.

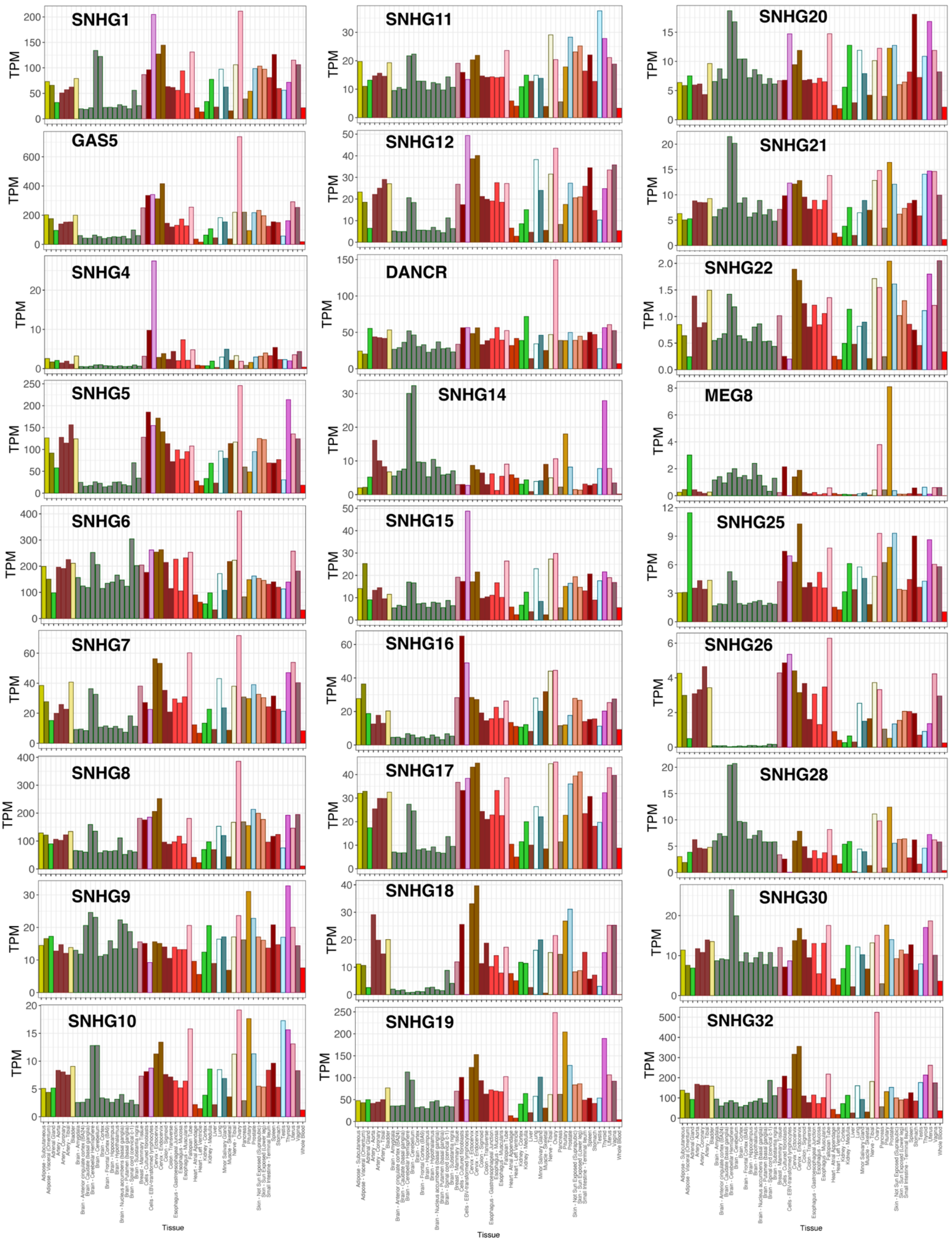

Appendix Figure S2. SNHGs expression across human tissues. Expression scores in Transcripts Per Million (TPM) were obtained from the GTEx V8 data.

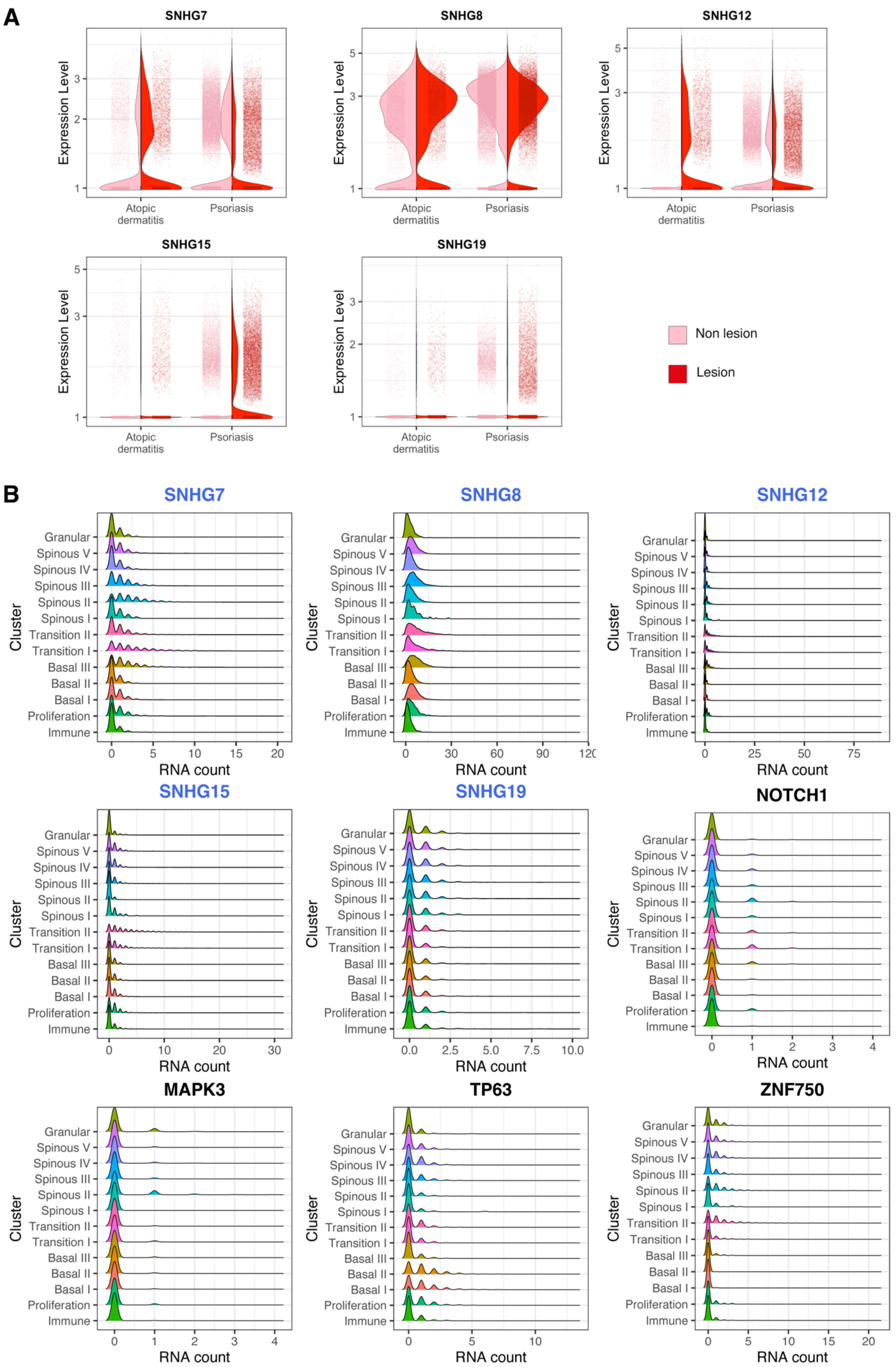
A
SNHG7
SNHG8
SNHG12
SNHG15
SNHG19
Expression Level
Atopic dermatitis
Psoriasis
Non lesion
Lesion
B
SNHG7
SNHG8
SNHG12
SNHG15
SNHG19
NOTCH1
MAPK3
TP63
ZNF750
Cluster
Granular
Spinous V
Spinous IV
Spinous III
Spinous II
Spinous I
Transition II
Transition I
Basal III
Basal II
Basal I
Proliferation
Immune
RNA count

Appendix Figure S3. Regulation and expression of epidermal SNHG in skin scRNA-seq datasets. (**A**) Expression of five epidermal SNHGs in scRNA-seq data from the lesional and non-lesional areas of atopic dermatitis, and psoriasis-affected tissues. (**B**) Estimation of copy number/cell of epidermal SNHGs at various stages of differentiation in scRNA-seq data from healthy skin. The copy number of mRNAs for known keratinocyte signalling mediators (MAPK3, NOTCH1) or transcription factors (TP63, ZNF750) are also shown for reference.

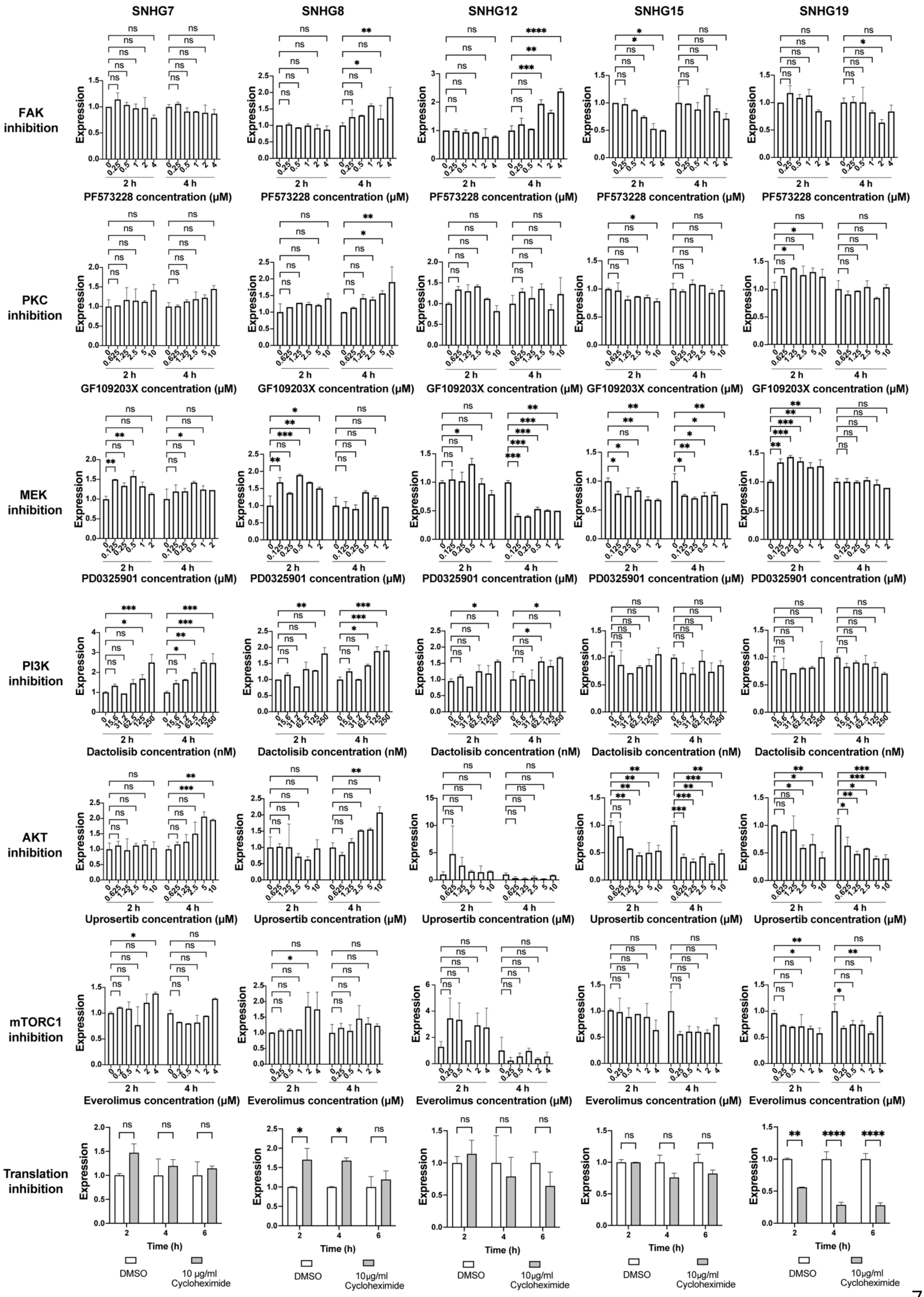
SNHG7
SNHG8
SNHG12
SNHG15
SNHG19
FAK inhibition
PKC inhibition
MEK inhibition
PI3K inhibition
AKT inhibition
mTORC1 inhibition
Translation inhibition
Expression
2 h
4 h
PF573228 concentration (µM)
GF109203X concentration (µM)
PD0325901 concentration (µM)
Dactolisib concentration (nM)
Uprosertib concentration (µM)
Everolimus concentration (µM)
Time (h)
DMSO
10 µg/ml Cycloheximide

Appendix Figure S4. Epidermal SNHGs expression is controlled by multiple signalling pathways. Changes in the expression of five epidermally expressed SNHGs in primary human keratinocytes in response to treatment with increasing doses of different pathway inhibitors for 2 h or 4 h. Data shown are mean +/- SD. Ordinary one-way ANOVA and Dunnett's multiple comparisons test, $n \geq 2$. **** $p < 0.0001$, *** $p < 0.001$, ** $p < 0.01$, * $p < 0.05$.

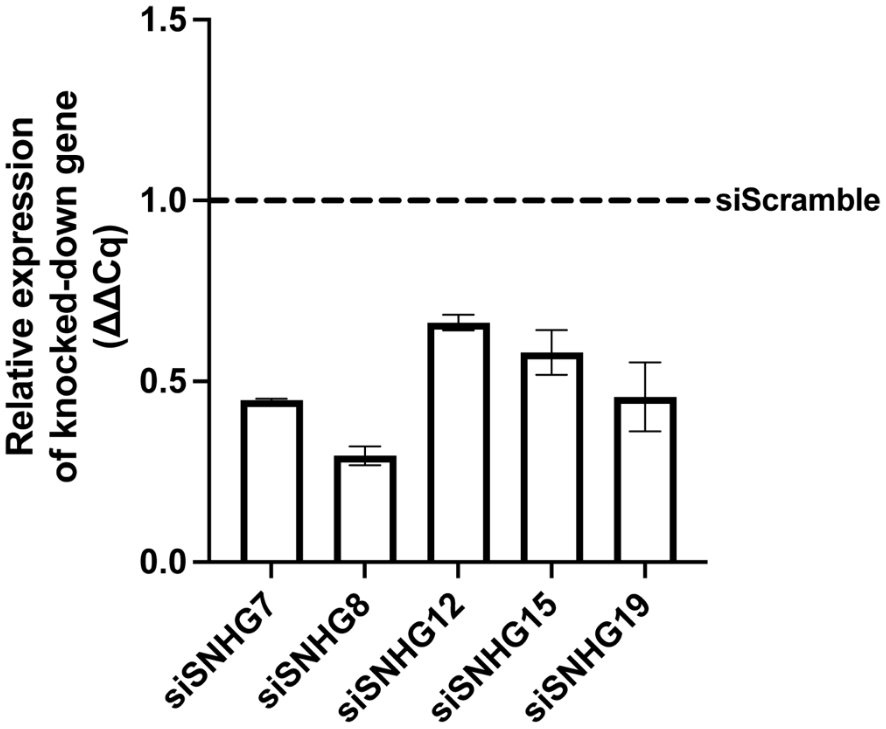

Appendix Figure S5. SNHG knockdown efficiency. Expression levels of epidermal SNHGs 24 h post-transfection with siRNA in the clonogenicity assays shown in Fig. 2E.

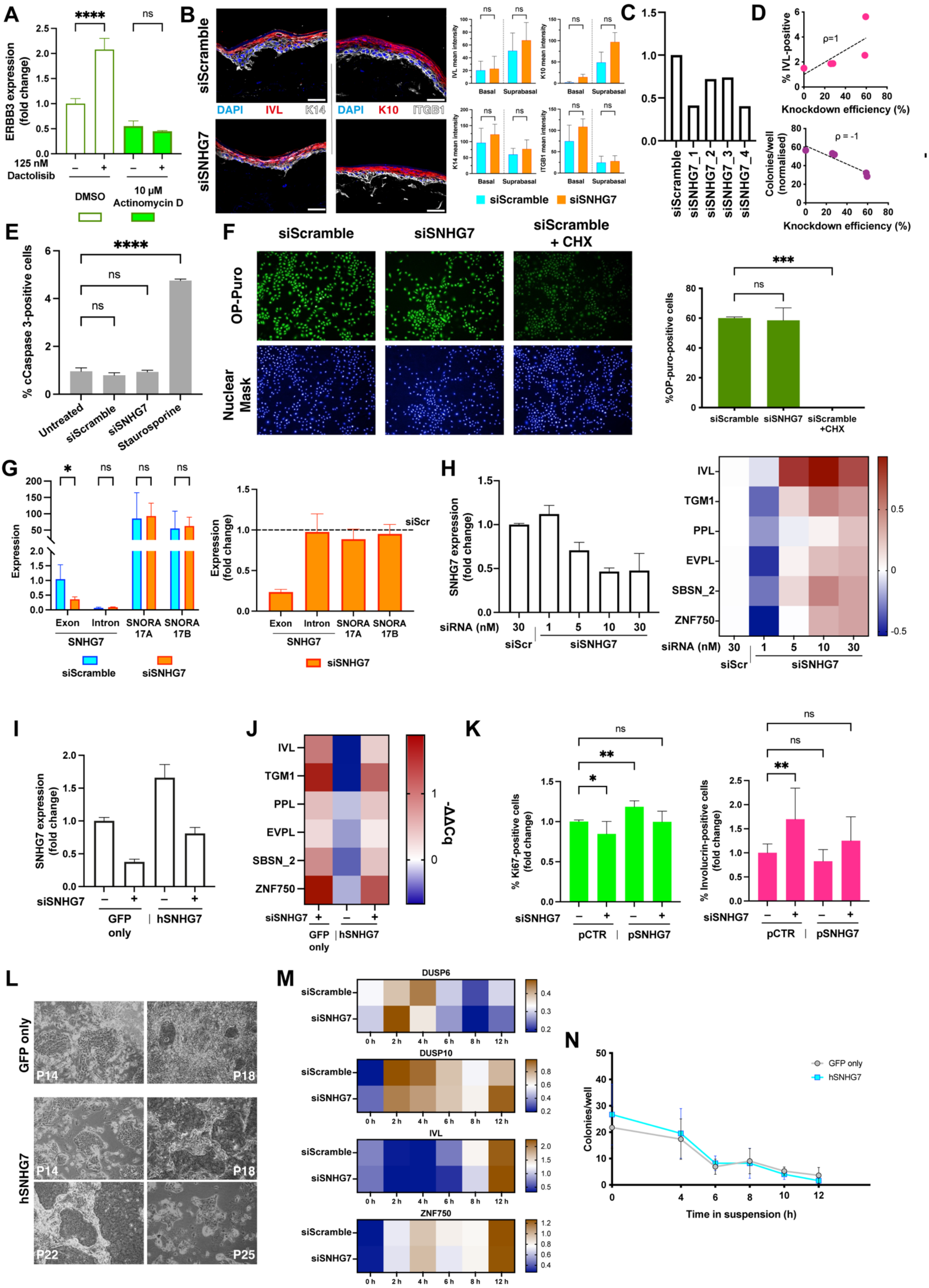
A
ERBB3 expression (fold change)
125 nM Dactolisib
DMSO
10 µM Actinomycin D
****
ns
B
siScramble
siSNHG7
DAPI IVL K14
DAPI K10 ITGB1
IVL mean intensity
K10 mean intensity
K14 mean intensity
ITGB1 mean intensity
Basal
Suprabasal
siScramble siSNHG7
C
siScramble
siSNHG7_1
siSNHG7_2
siSNHG7_3
siSNHG7_4
D
% IVL-positive
ρ=1
Knockdown efficiency (%)
Colonies/well (normalised)
ρ = -1
Knockdown efficiency (%)
E
% cCaspase 3-positive cells
Untreated
siScramble
siSNHG7
Staurosporine
F
siScramble
siSNHG7
siScramble + CHX
OP-Puro
Nuclear Mask
%OP-puro-positive cells
siScramble+CHX
G
Expression
Exon
Intron
SNORA 17A
SNORA 17B
SNHG7
siScramble
siSNHG7
Expression (fold change)
siScr
H
SNHG7 expression (fold change)
siRNA (nM)
siScr
siSNHG7
IVL
TGM1
PPL
EVPL
SBSN_2
ZNF750
I
SNHG7 expression (fold change)
siSNHG7
GFP only
hSNHG7
J
-ΔΔCq
K
% Ki67-positive cells (fold change)
% Involucrin-positive cells (fold change)
pCTR
pSNHG7
L
GFP only
hSNHG7
P14
P18
P22
P25
M
DUSP6
DUSP10
IVL
ZNF750
0 h 2 h 4 h 6 h 8 h 12 h
N
Colonies/well
GFP only
hSNHG7
Time in suspension (h)

Appendix Figure S6. SNHG7 lncRNA affects epidermal stem cell proliferation and differentiation. (**A**) ERBB3 expression after 4 h PI3K inhibition with or without prior 1 h treatment with a transcription inhibitor. n = 5 independent treatments. (**B**) Epidermal differentiation marker staining of epidermis reconstituted by control or SNHG7 knockdown cells and quantification. n ≥ 4 images taken from four DEDs/condition. (**C**-**D**) Deconvolution of the siRNA pool targeting SNHG7. Transfection efficiency of the individual siRNAs (**C**) and correlation between knockdown level and Involucrin expression (**D**, top) or clonogenic capacity (**D**, bottom). (**E**) Effect of SNHG7 knockdown on apoptosis. Quantification of the percentage of cells staining positive for cleaved Caspase 3. n = 2 independent transfections/treatments. (**F**) Effect of SNHG7 knockdown on translation. Representative images (left panels) and quantification (right bar plot) of keratinocytes stained with OP-Puro in control conditions, after SNHG7 knockdown and after treatment with translation inhibitor cycloheximide (CHX). n = 2 independent treatments/transfections. (**G**) Effect of siSNHG7 treatment on different transcripts arising from the locus. Shown are expression levels relative to reference genes (left) and change in expression after knockdown relative to control (right). n = 2 independent transfections. (**H**) Titration of siSNHG7 to identify the minimal effective concentration. Knockdown efficiency at 48 h post transfection (left) and differentiation marker expression heatmap (right). (**I**-**K**) Rescue of the SNHG7 knockdown phenotype by lncRNA overexpression. Shown are SNHG7 lncRNA expression levels in all samples (**I**), differentiation marker expression 72 h post-transfection relative to control (**J**), and quantification of differentiation and proliferation marker staining 96 h post-transfection (**K**). n = 8 independent transfections. (**L**) Representative images of cultured keratinocytes overexpressing SNHG7 or GFP only at different passages. Cells were transduced at the same time and were subcultured seeding the same number of cells at each passage. (**M**) Time course of commitment (DUSP6, DUSP10) and differentiation (ZNF750, IVL) marker expression after suspension-induced differentiation of control and SNHG7 knockdown cells. pCTR, control plasmid; pSNHG7, overexpression plasmid. (**N**) Suspension-induced differentiation time course of control and SNG7-overexpressing keratinocytes. Scale bars, 50 μm. Data shown in all bar plots are mean +/- SD. (**A)** Ordinary one-way ANOVA and Šidák's multiple comparisons test. (**B**) Kruskal-Wallis test and Dunn's multiple comparisons test. (**E**) (**F**) and (**K**) Ordinary one-way ANOVA and Dunnett's multiple comparisons test. (**G**) Multiple unpaired two-tailed t-tests. **** $p < 0.0001$, *** $p < 0.001$, ** $p < 0.01$, * $p < 0.05$.

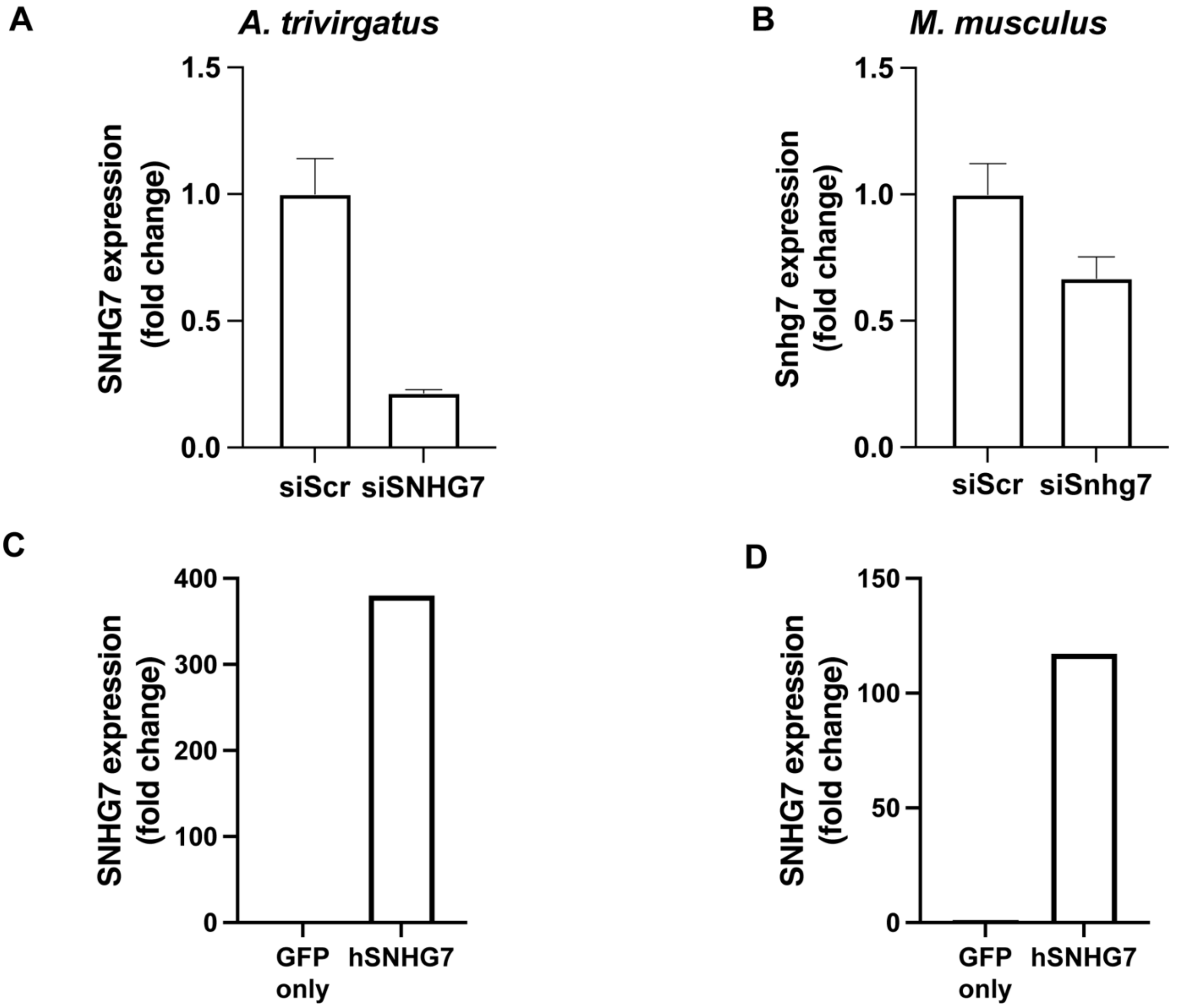

Appendix Figure S7. SNHG7 knockdown and overexpression efficiency in *A. trivirgatus* and *M. musculus* cells. (**A**-**B**) Expression of endogenous SNHG7 after 48h siRNA transfection in *A. trivirgatus* (**A**) or *M. musculus* (**B**) keratinocytes. (**C-D**) Expression of human SNHG7 in *A. trivirgatus* (**C**) or *M. musculus* (**D**) keratinocytes after stable transfection with an overexpression plasmid.

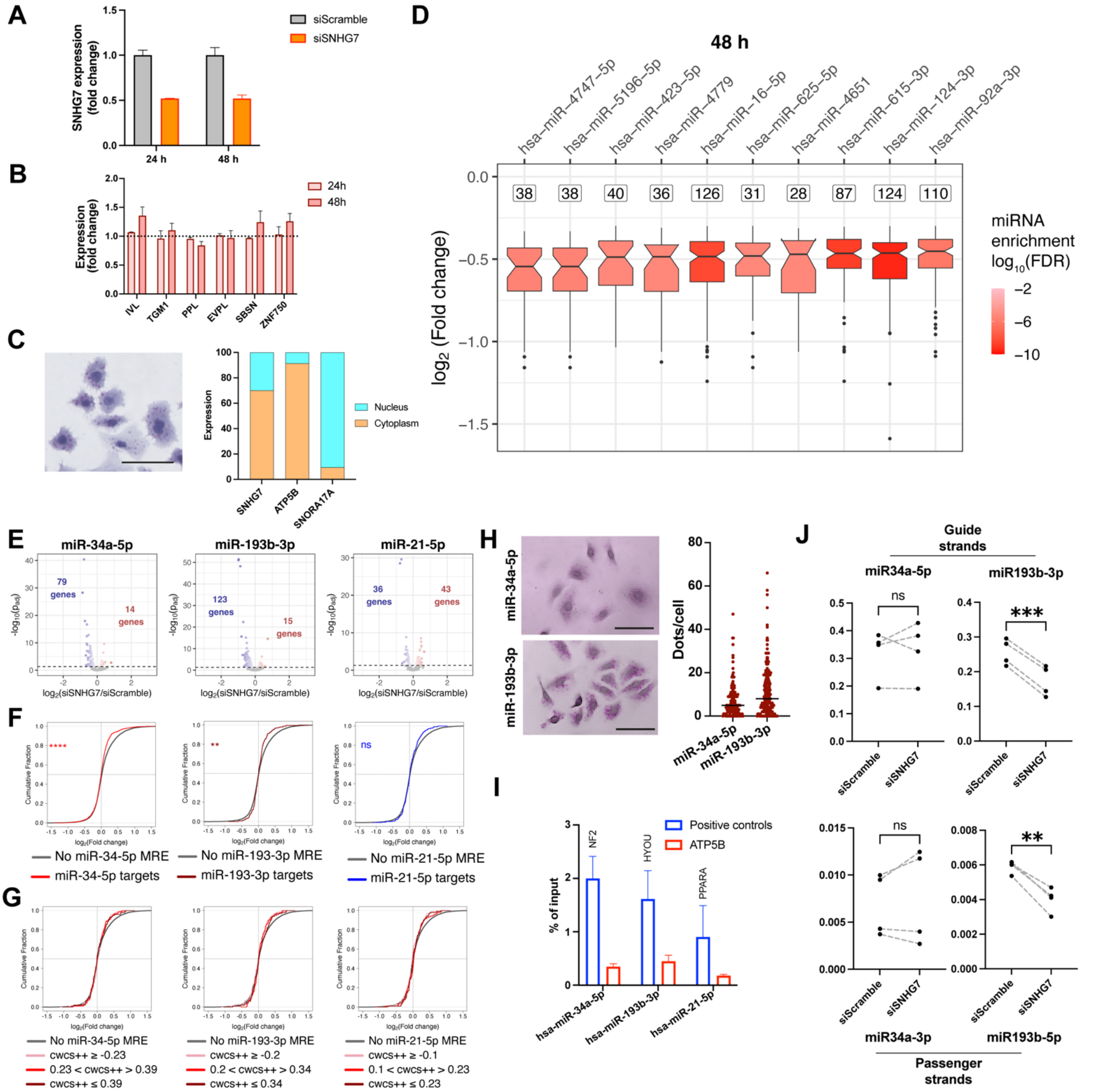

Appendix Figure S8. SNHG7 lncRNA affects the expression of targets of interacting miRNAs. (**A**) Knockdown efficiency in the RNA-seq. (**B**) Expression variation of selected differentiation markers in the RNA-seq. (**C**) Intracellular distribution of SNHG7 assessed by smRNA cytochemistry (left) or subcellular fractionation (right). Subcellular distributions of a mRNA (ATP5B) and a snoRNA (SNORA17A) are used as controls and references. (**D**) miRNA response element enrichment in significantly (p_{adj} < 0.05) downregulated genes 48 h post-transfection. The number of target genes for each miRNA is shown above the box plot of their differential expression. miRNAs are sorted based on the median downregulation of their target genes. The colour of the boxes indicates the significance of the enrichment. (**E**) Validated miRNA target genes in the siSNHG7 RNAseq data. Volcano plots show all validated target genes for the two candidate miRNAs (miR-34a-5p and miR-193b-3p), and a control miRNA (miR-21-5p). (**F**) Cumulative distributions of the gene expression change between control and knockdown cells for predicted targets of miR-34a (red, left), miR-193b (dark red, centre) or miR-21 (blue, right) compared to transcripts that do not contain the respective MREs (grey). Kolmogorov-Smirnov test. (**G**) Same as (**F**) with targets for each miRNA

split in equally sized bins of miRNA affinity estimated by cumulative weighted context++ score (cwcs++). (**H**) *In situ* hybridisation cytochemistry and quantification of the expression of two candidate miRNAs in primary human keratinocytes. Scale bars, 50 μm. Lines indicate the median. (**I**) Enrichment of control genes after pulldown of biotinylated miRNAs. Genes containing MREs for the indicated miRNAs are used as positive controls for the pulldown and are indicated above the bars. ATP5B was used as a negative control as it does not contain MREs for any of the miRNAs. (**J**) Variation in the levels of guide and passenger strands of candidate miRNAs after SNHG7 knockdown. Paired two-tailed t-tests. Pairs of points (connected by dotted grey lines) represent individual experiments. Data shown in all bar plots are mean +/- SD. **** $p < 0.0001$, *** $p < 0.001$, ** $p < 0.01$.

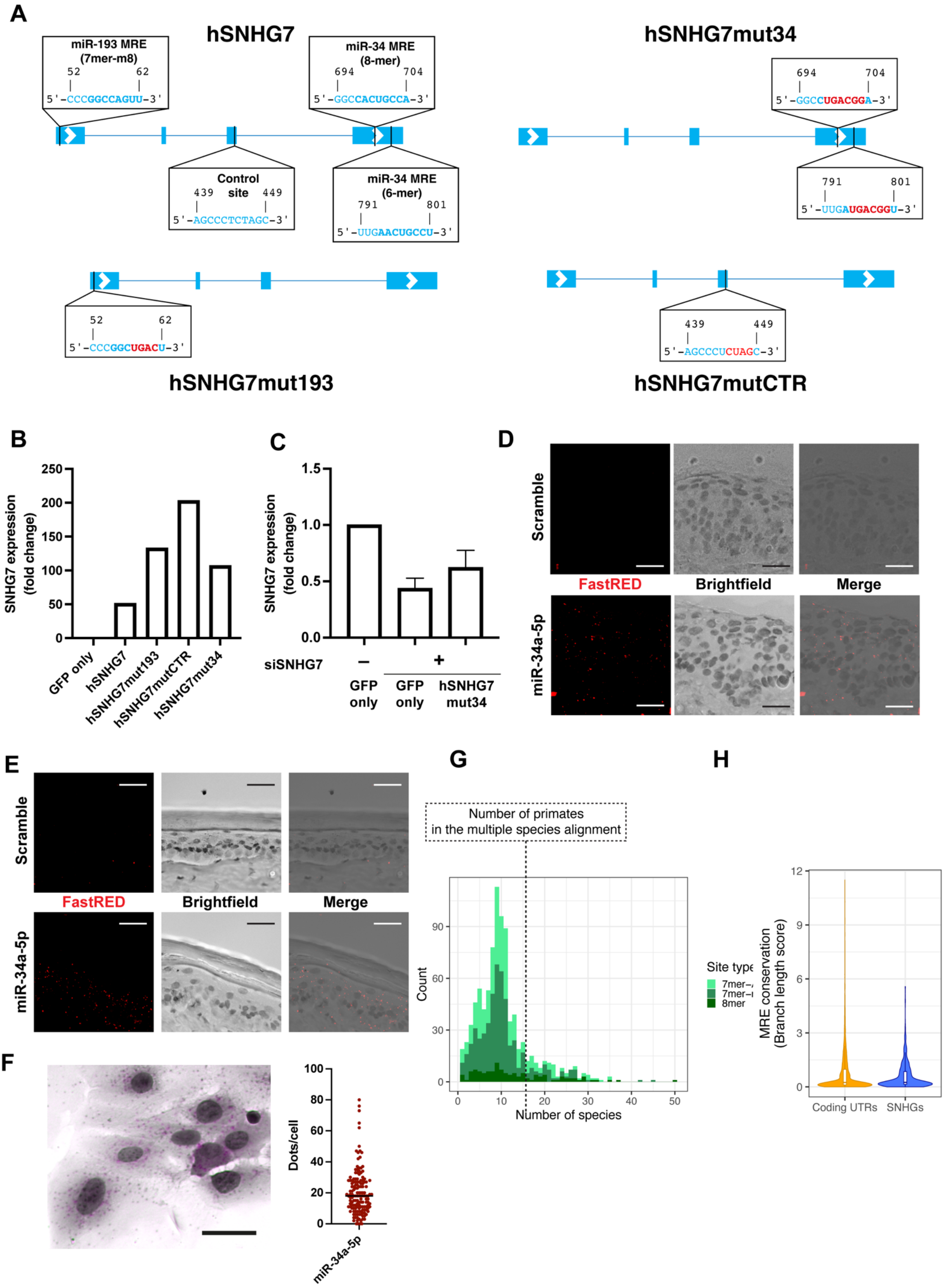
A
hSNHG7
miR-193 MRE
(7mer-m8)
52 62
5'-CCCGGCCAGUU-3'
miR-34 MRE
(8-mer)
694 704
5'-GGCCACUGCCA-3'
Control site
439 449
5'-AGCCCTCTAGC-3'
miR-34 MRE
(6-mer)
791 801
5'-UUGAACUGCCU-3'
hSNHG7mut34
694 704
5'-GGCCUGACGGA-3'
791 801
5'-UUGAUGACGGU-3'
52 62
5'-CCCGGCUGACU-3'
hSNHG7mut193
439 449
5'-AGCCCUCUAGC-3'
hSNHG7mutCTR
B
SNHG7 expression
(fold change)
250
200
150
100
50
0
GFP only
hSNHG7
hSNHG7mut193
hSNHG7mutCTR
hSNHG7mut34
C
SNHG7 expression
(fold change)
1.5
1.0
0.5
0.0
siSNHG7
–
+
GFP only
GFP only
hSNHG7 mut34
D
Scramble
FastRED
Brightfield
Merge
miR-34a-5p
E
Scramble
FastRED
Brightfield
Merge
miR-34a-5p
F
100
80
60
40
20
0
Dots/cell
miR-34a-5p
G
Number of primates
in the multiple species alignment
Count
90
60
30
0
0 10 20 30 40 50
Number of species
H
Site type
8mer
12
9
6
3
0
MRE conservation
(Branch length score)
Coding UTRs
SNHGs

Appendix Figure S9. Evolution of SNHG-MRE interactions. (**A**) Detailed schematic of the mutant transcripts used in overexpression/rescue experiments. (**B**) Expression of human SNHG7 mutants in *A. trivirgatus* keratinocytes after knockdown stable transduction with an overexpression plasmid. (**C**) Expression of SNHG7 in human keratinocytes after knockdown in keratinocytes stably transduced with a control (GFP only) or SNHG7 mutant overexpression plasmid. (**D**) *In situ* hybridisation histochemistry of miR-34-5p in human skin sections. (**E**) *In situ* hybridisation histochemistry of miR-34-5p in mouse skin sections. (**F**) *In situ* hybridisation cytochemistry and quantification of the expression of miR-34-5p in primary mouse keratinocytes. Line indicates the median. (**G**) Distribution of the MREs present on human SNHGs according to the number of species that share them. MREs are broken down into the three different types of seed binding. A dashed line marks the number of primate species in the multiple alignment used for this analysis, indicating that all MREs shared by a larger number of species are conserved beyond the primate lineage. MREs shared by a smaller number of species can either be conserved only among primates or be conserved beyond the primate lineage but lost in one or more primate species. (**H**) Conservation of MREs for deeply conserved miRNAs found in SNHGs and in a set of 250 3'UTRs of coding genes. Scale bars, 25 μm. Data shown in all bar plots are mean +/- SD.
