# Supplementary figures and images for: Neutral evolution of snoRNA Host Gene long non-coding RNA affects cell fate control
Source: EMBO J. 2024 Jul 25;43(18):4049–67. doi: 10.1038/s44318-024-00172-8 (PMC11405852; doi:10.1038/s44318-024-00172-8)

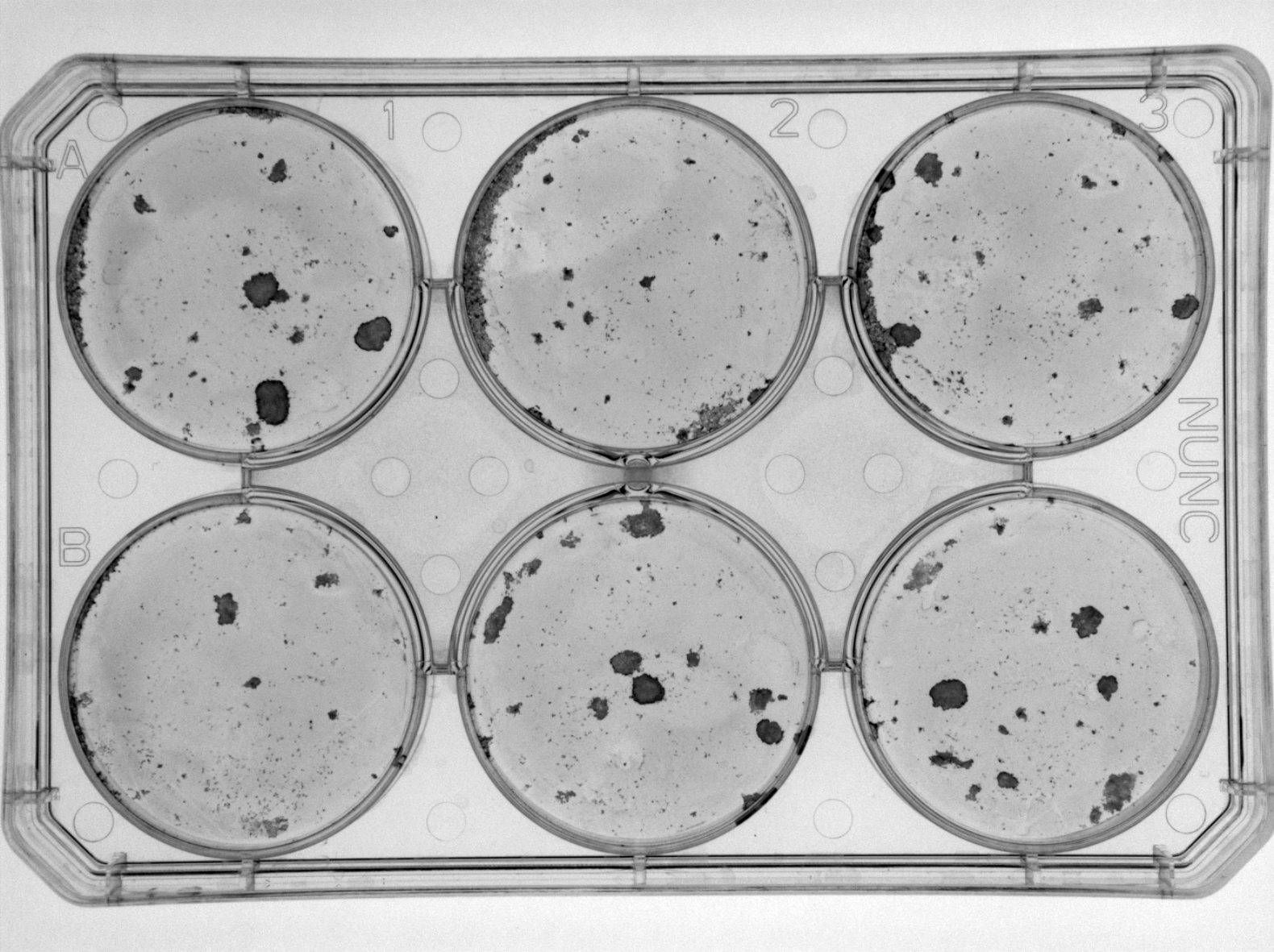

Supplement: Supplementary file 4 — Source data Fig. 2 [file 44318_2024_172_MOESM4_ESM.zip › Figure 2/2E/Fig.2E_siSNHG19.tif]

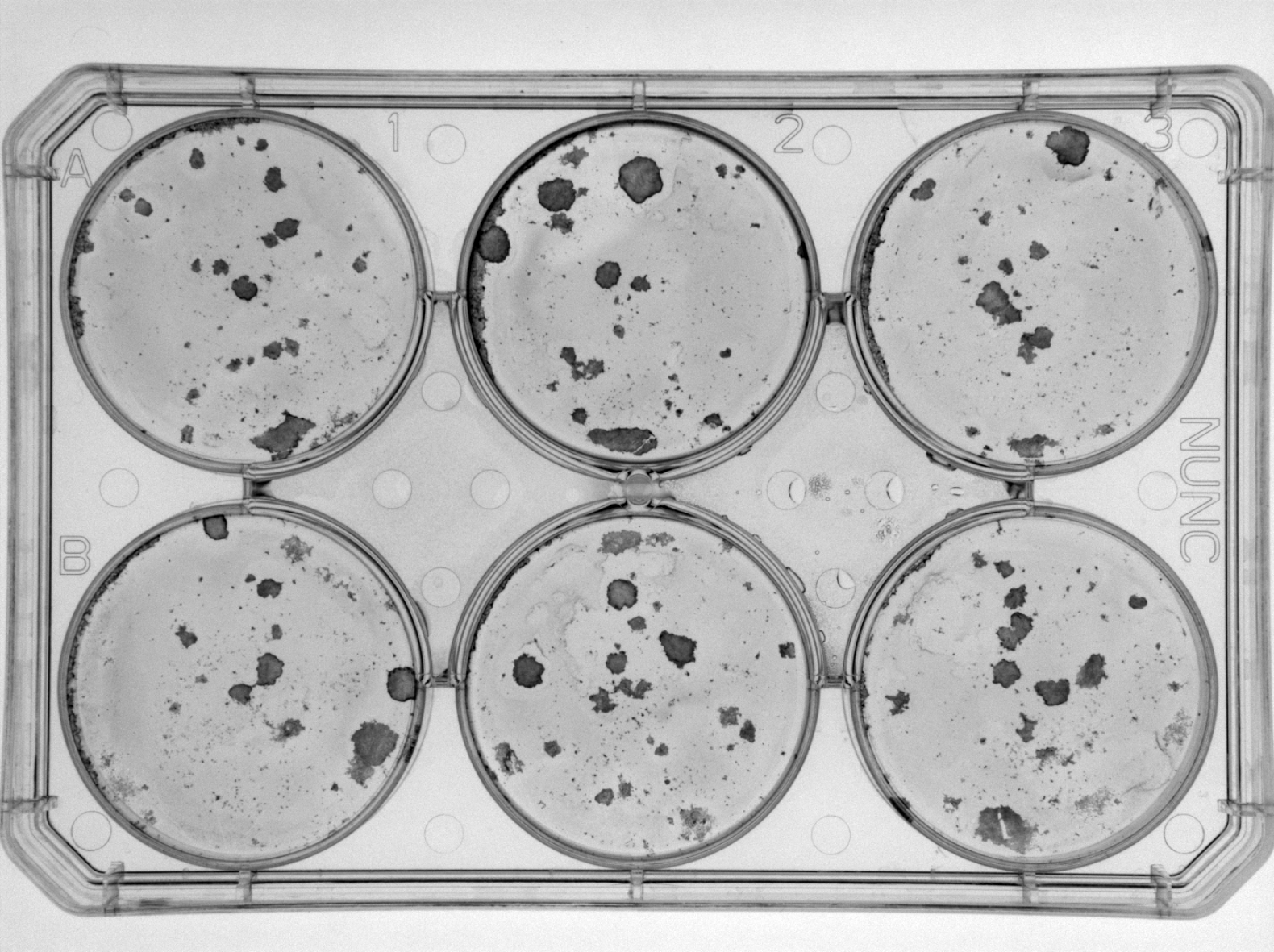

Supplement: Supplementary file 4 — Source data Fig. 2 [file 44318_2024_172_MOESM4_ESM.zip › Figure 2/2E/Fig.2E_siSNHG8.tif]

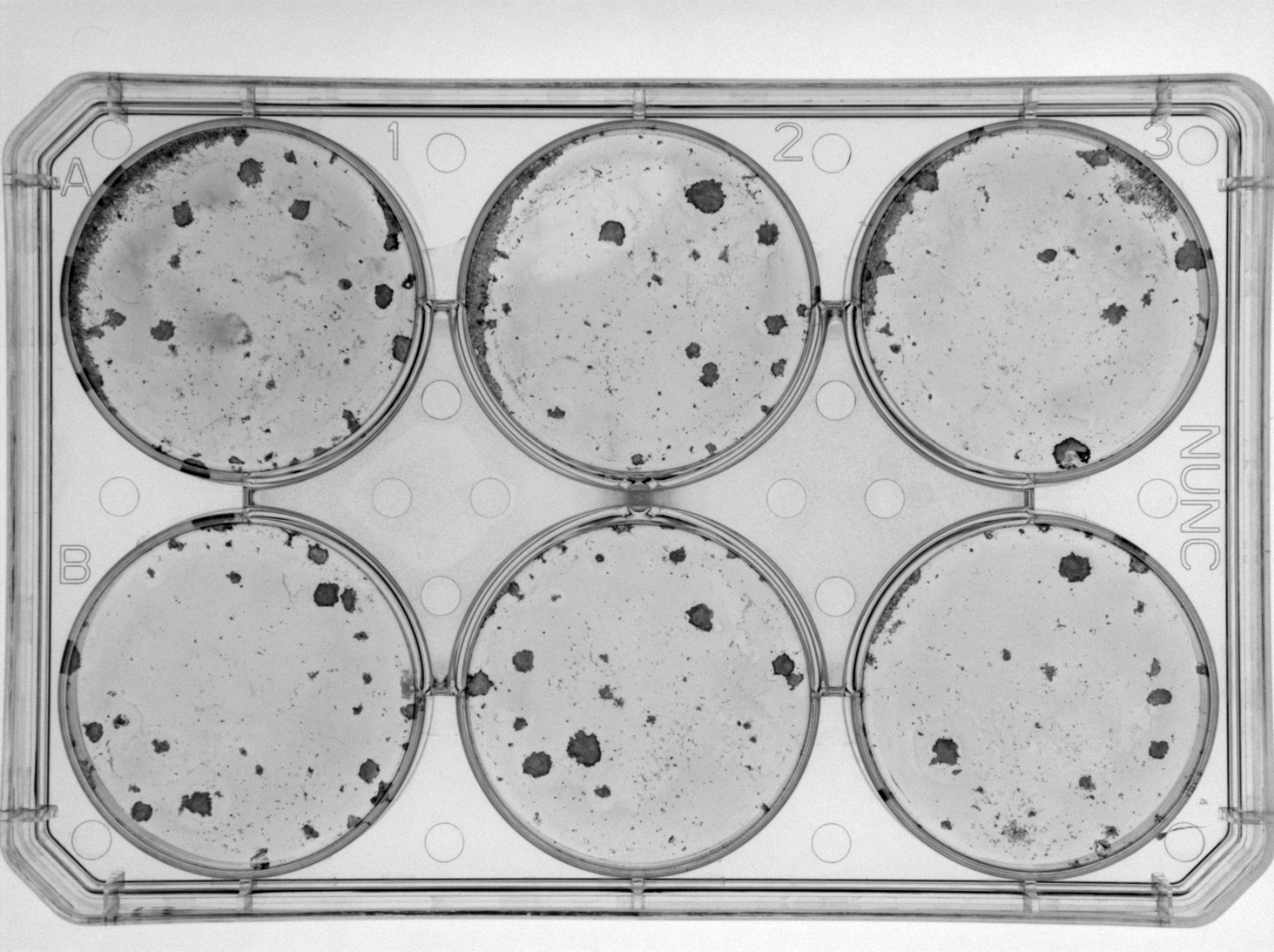

Supplement: Supplementary file 4 — Source data Fig. 2 [file 44318_2024_172_MOESM4_ESM.zip › Figure 2/2E/Fig.2E_siSNHG12.tif]

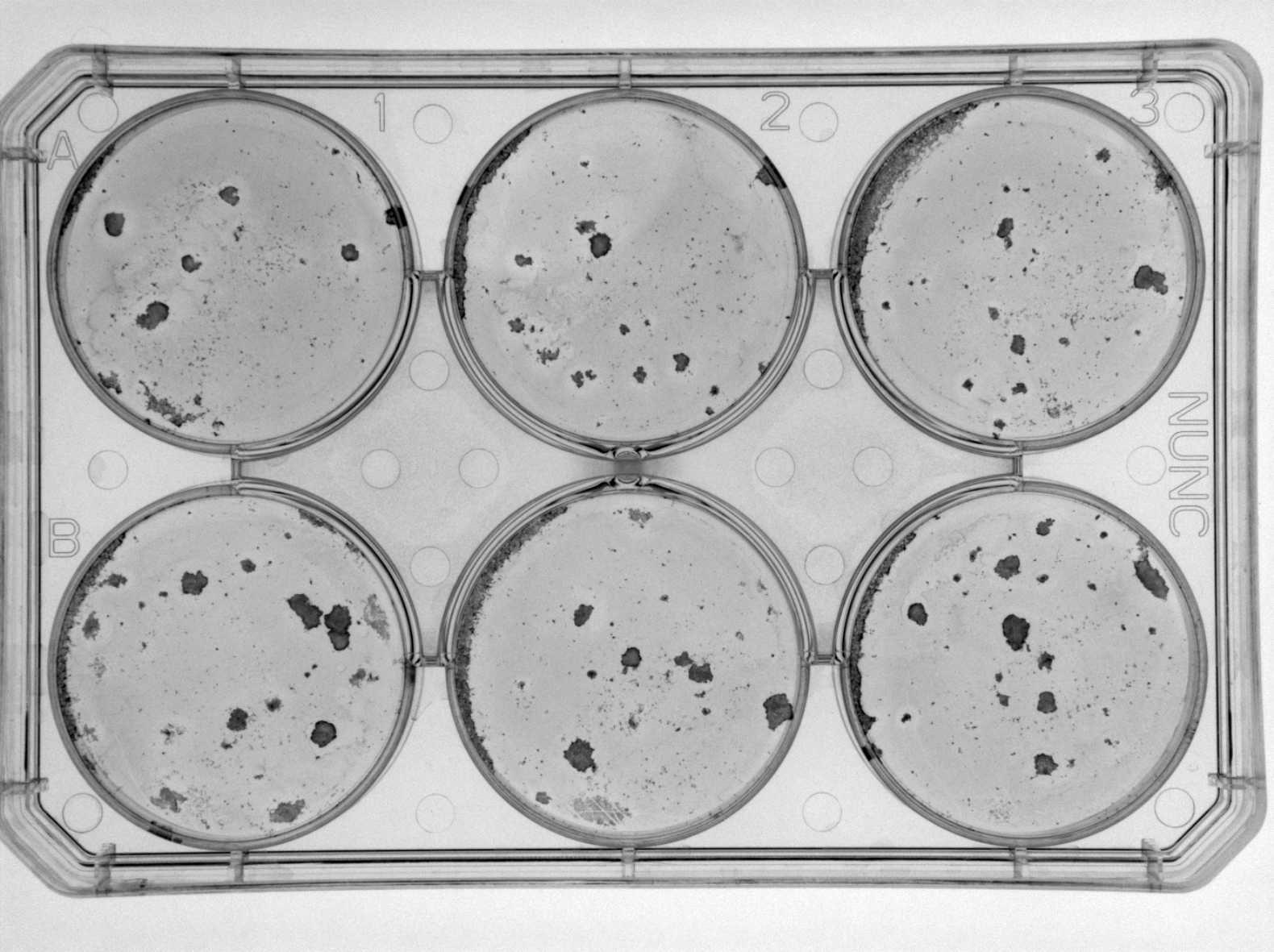

Supplement: Supplementary file 4 — Source data Fig. 2 [file 44318_2024_172_MOESM4_ESM.zip › Figure 2/2E/Fig.2E_siSNHG7.tif]

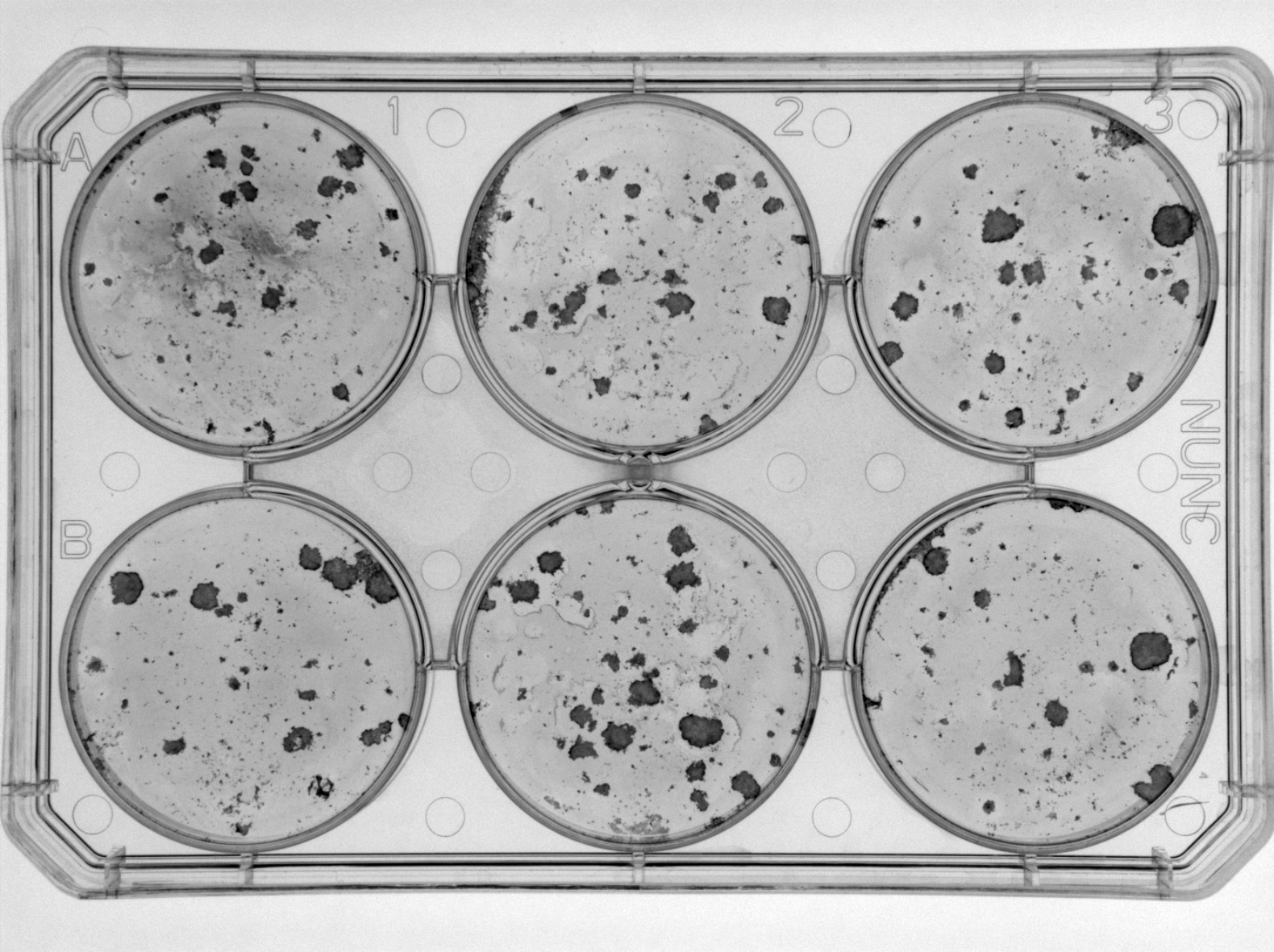

Supplement: Supplementary file 4 — Source data Fig. 2 [file 44318_2024_172_MOESM4_ESM.zip › Figure 2/2E/Fig.2E_siScramble.tif]

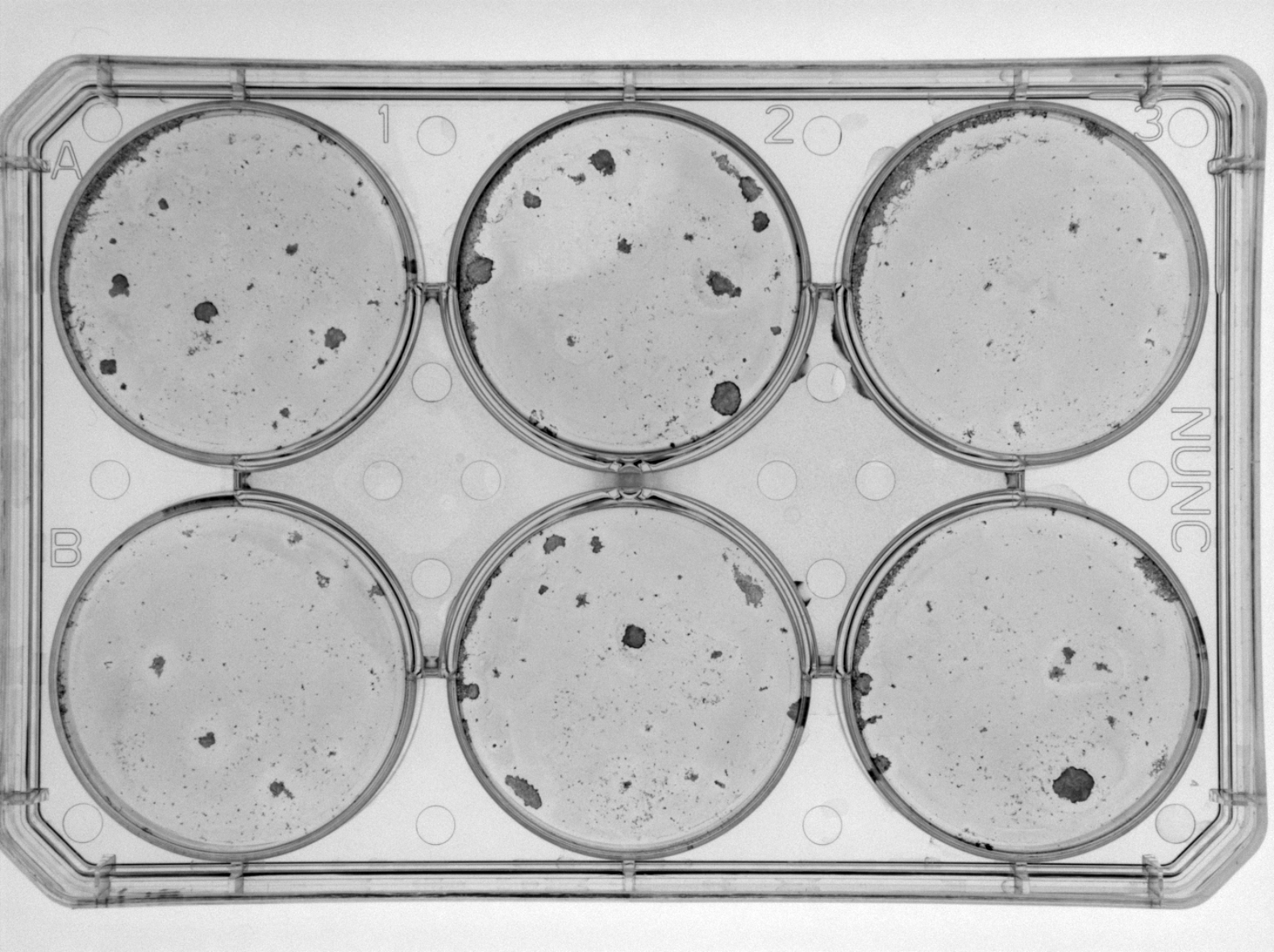

Supplement: Supplementary file 4 — Source data Fig. 2 [file 44318_2024_172_MOESM4_ESM.zip › Figure 2/2E/Fig.2E_siSNHG15.tif]

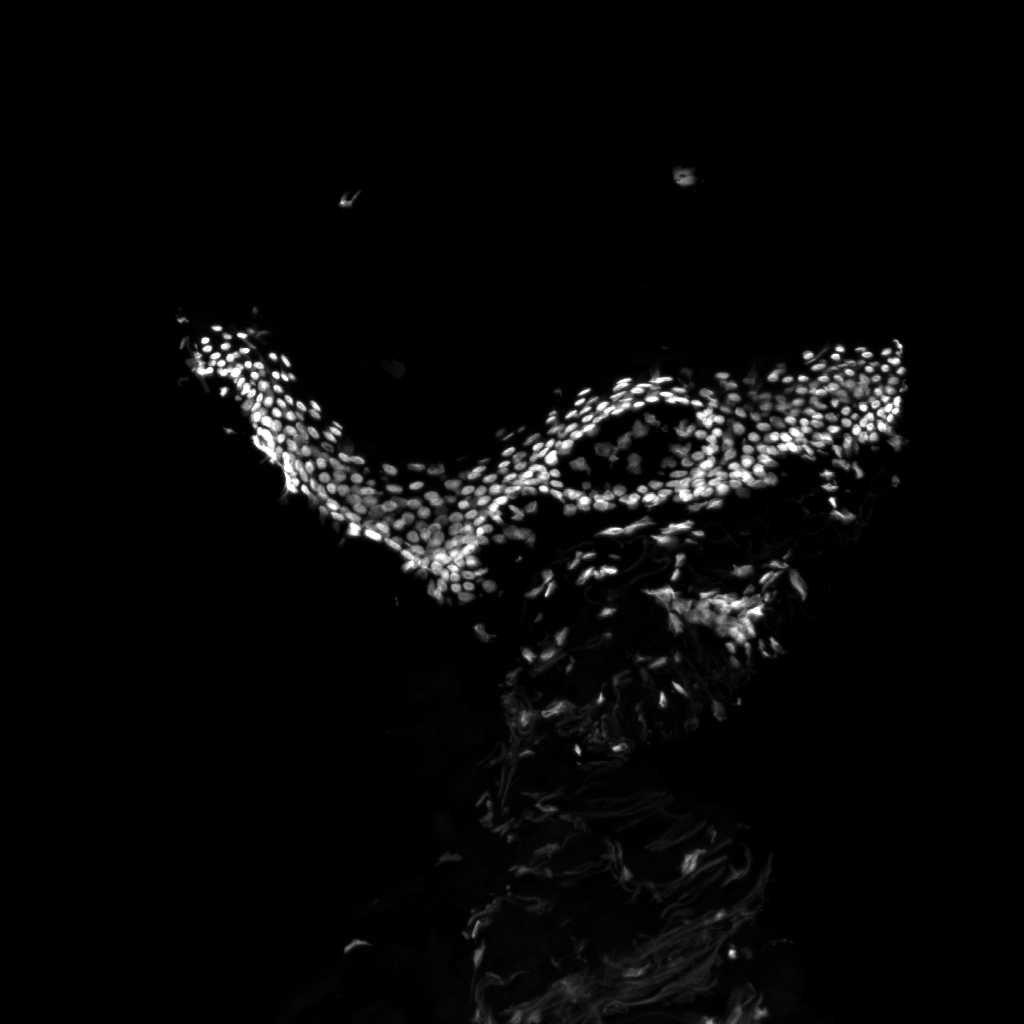

Supplement: Supplementary file 5 — Source data Fig. 3 [file 44318_2024_172_MOESM5_ESM.zip › Figure 3/3E/Fig.3E_Non Lesional.tif]

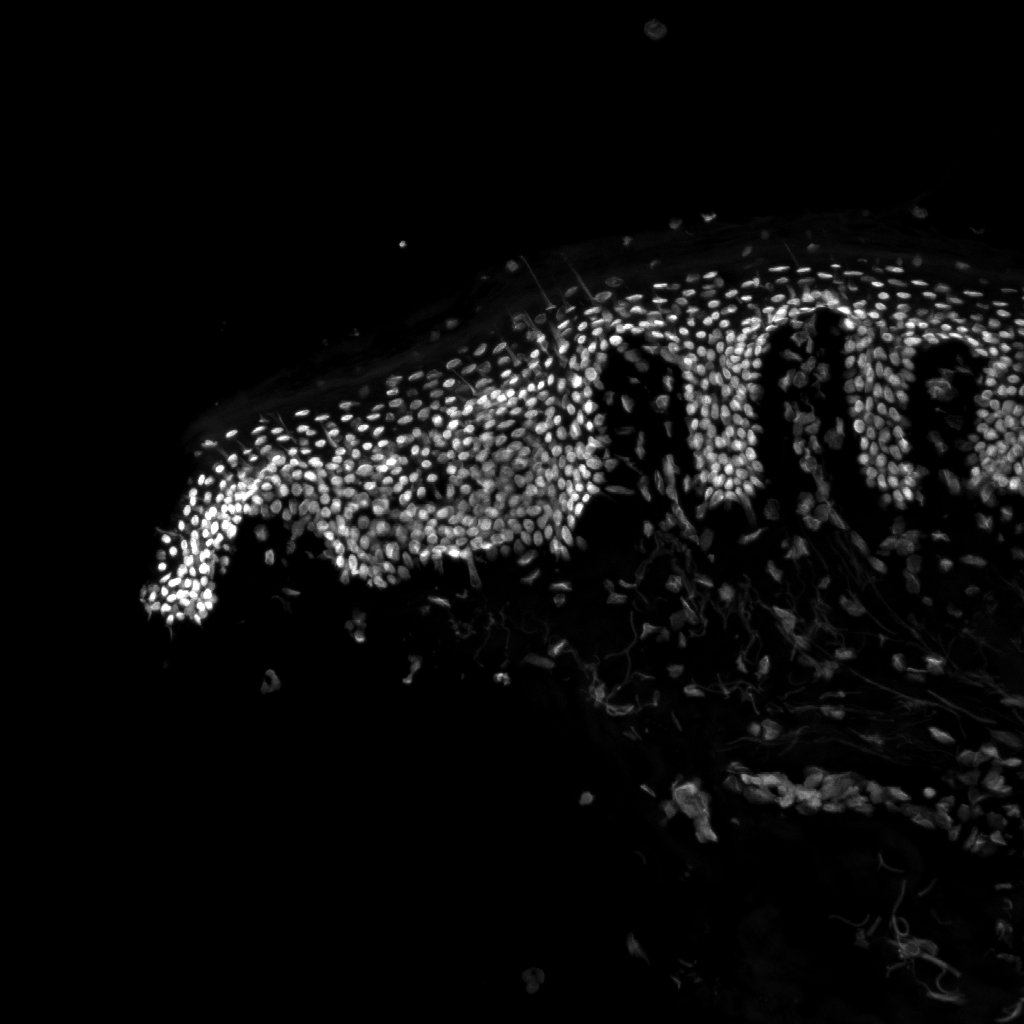

Supplement: Supplementary file 5 — Source data Fig. 3 [file 44318_2024_172_MOESM5_ESM.zip › Figure 3/3E/Fig.3E_Lesional.tif]

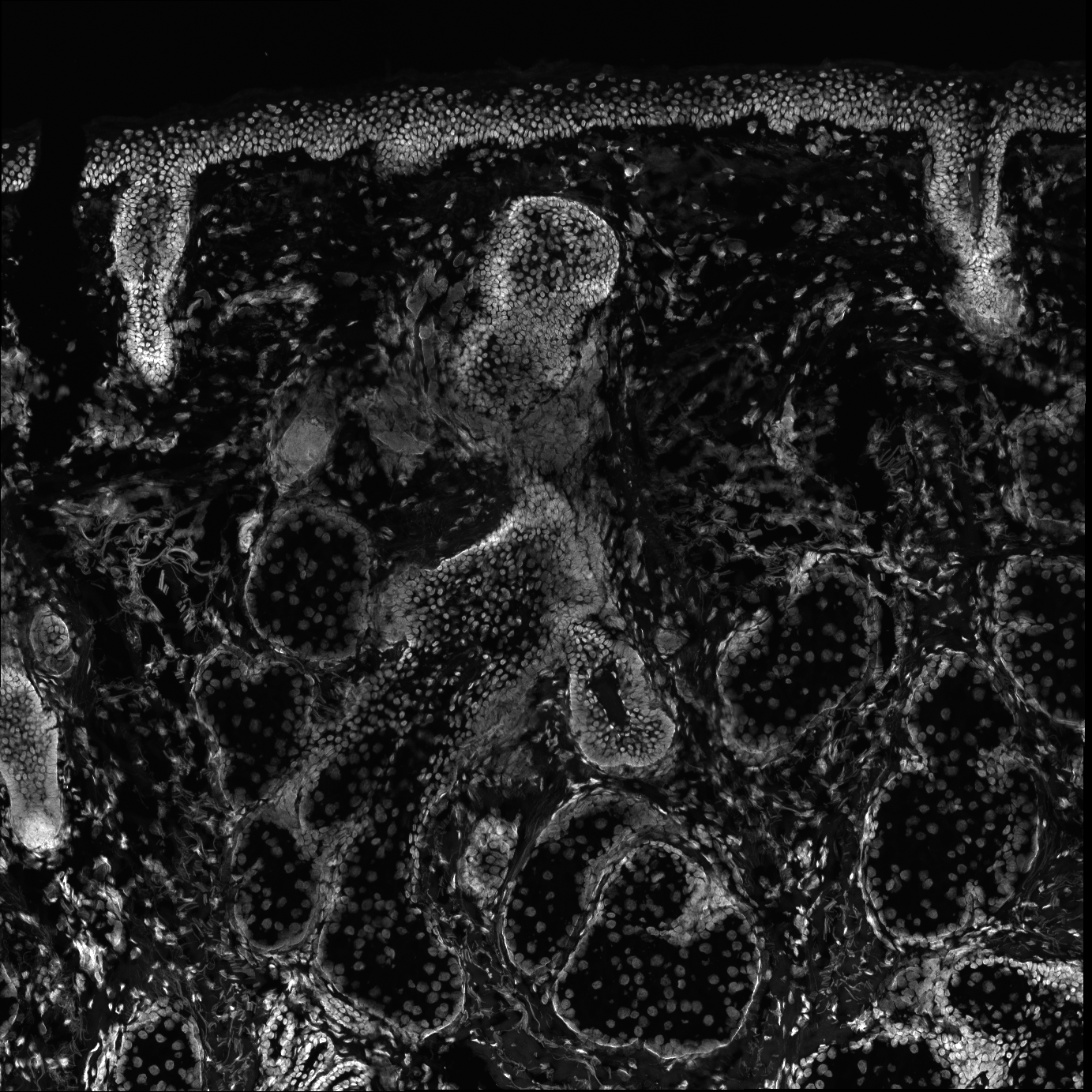

Supplement: Supplementary file 5 — Source data Fig. 3 [file 44318_2024_172_MOESM5_ESM.zip › Figure 3/3D/Fig.3D.tif]

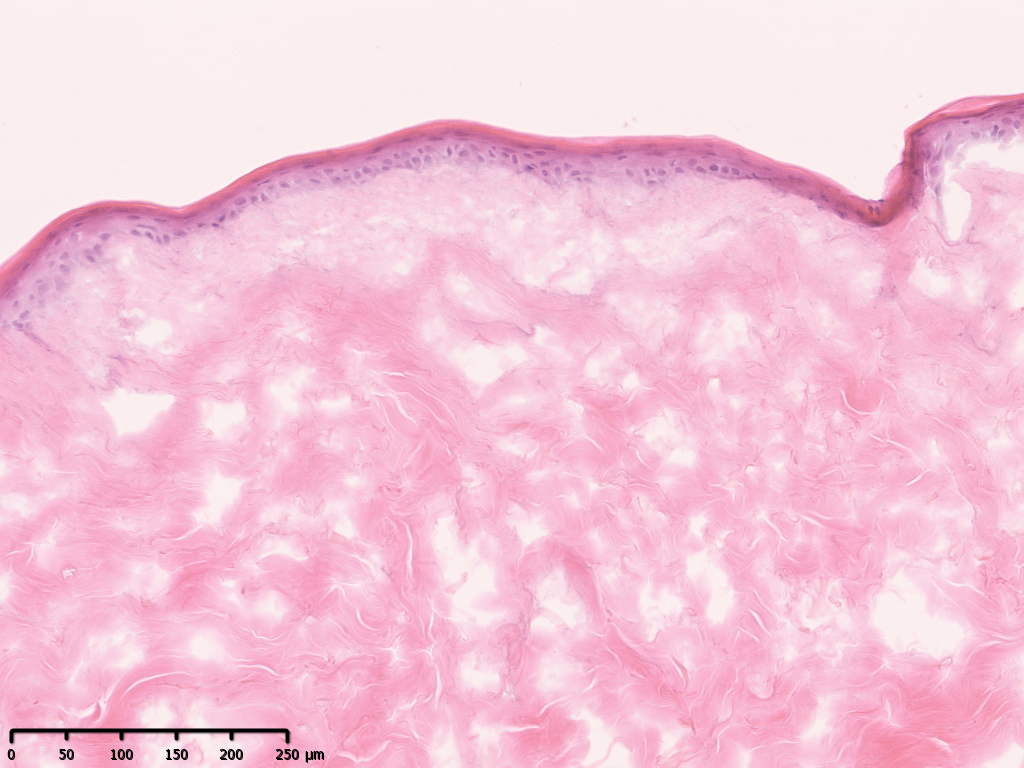

Supplement: Supplementary file 5 — Source data Fig. 3 [file 44318_2024_172_MOESM5_ESM.zip › Figure 3/3H/Fig.3H_siSNHG7.tif]

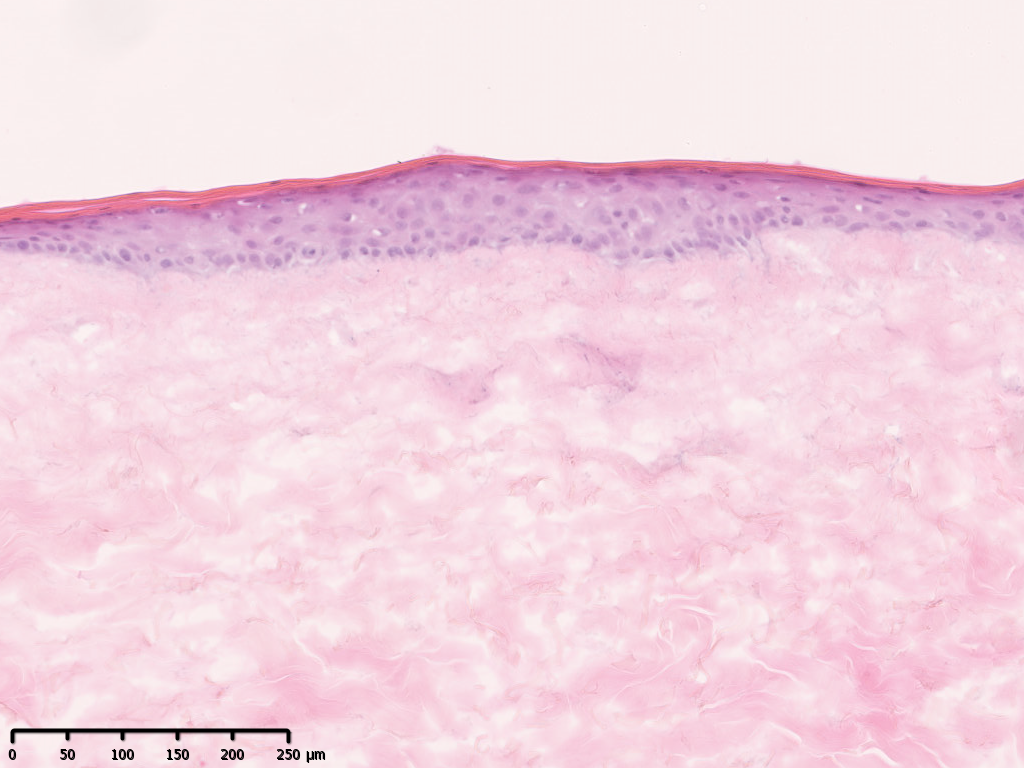

Supplement: Supplementary file 5 — Source data Fig. 3 [file 44318_2024_172_MOESM5_ESM.zip › Figure 3/3H/Fig.3H_siScramble.tif]

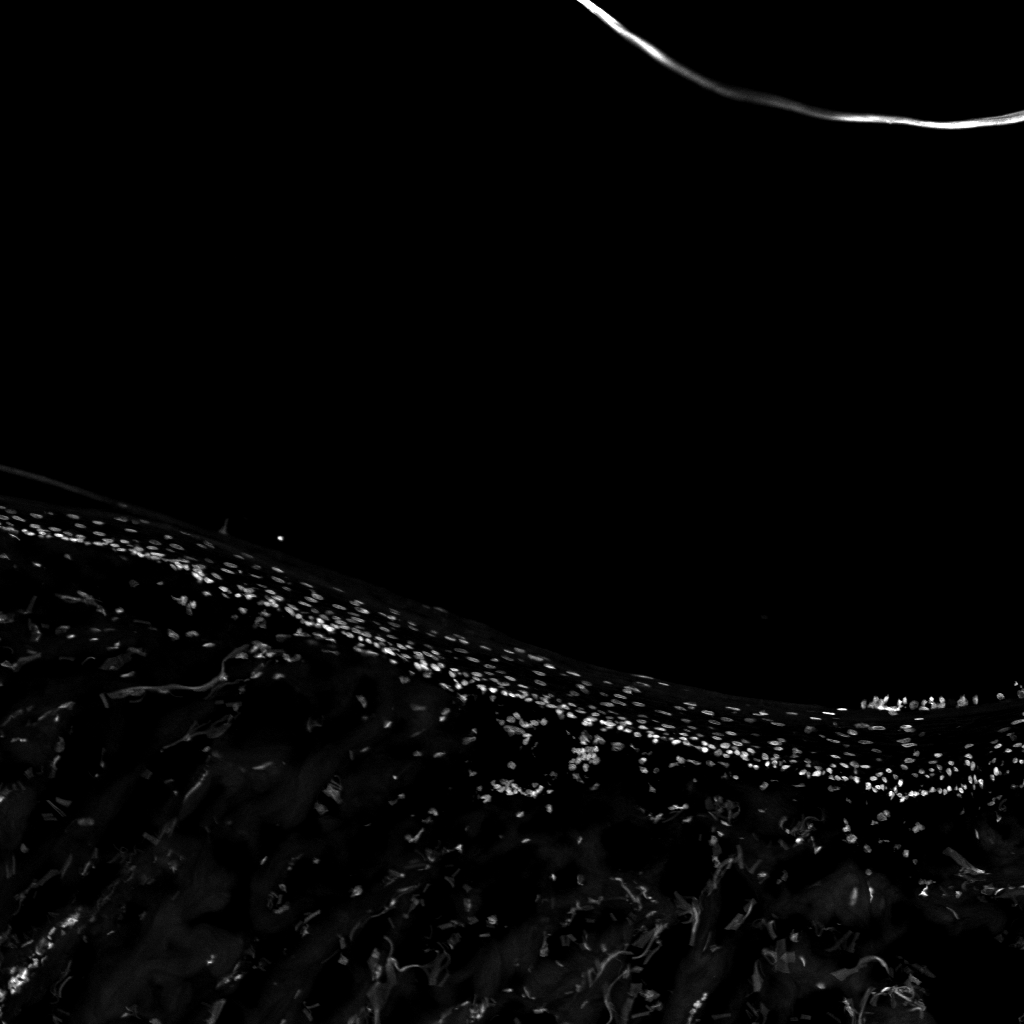

Supplement: Supplementary file 5 — Source data Fig. 3 [file 44318_2024_172_MOESM5_ESM.zip › Figure 3/3F/Fig.3F_Wound.tif]

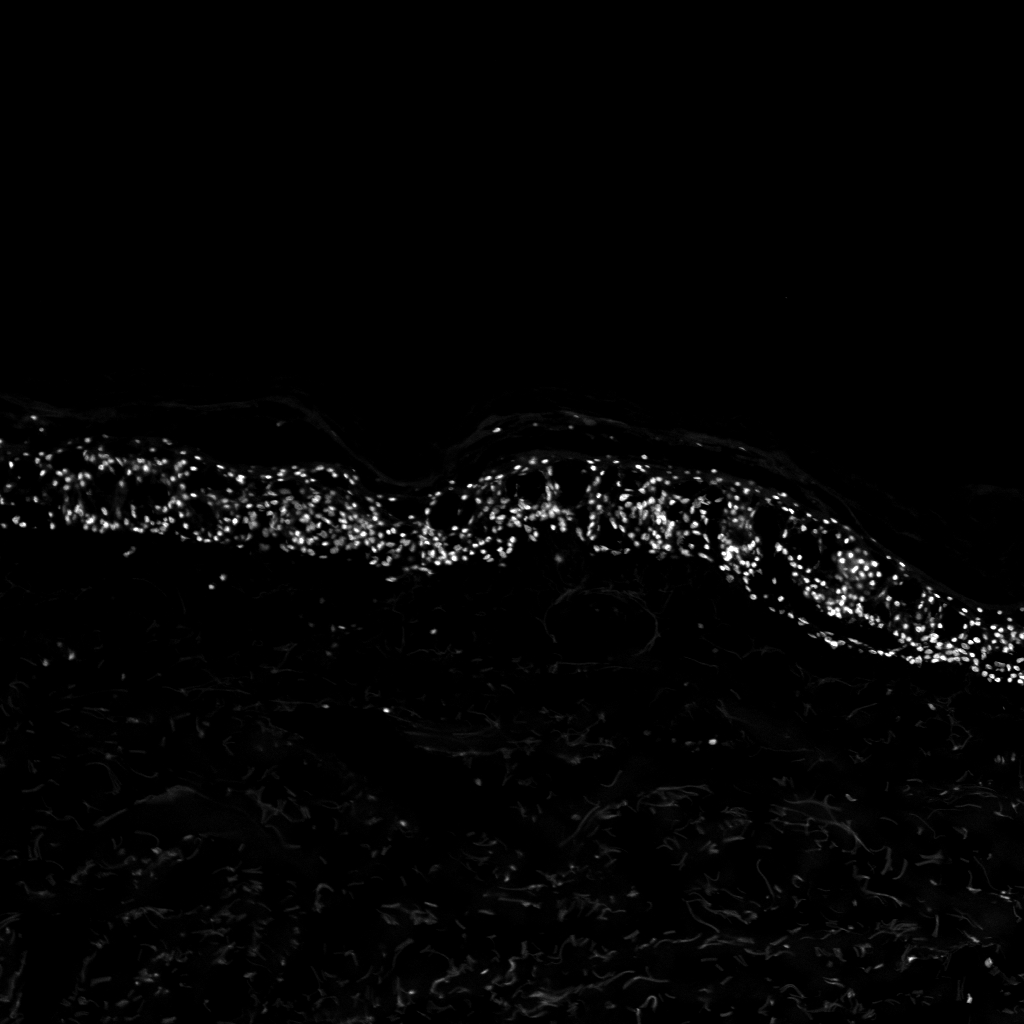

Supplement: Supplementary file 5 — Source data Fig. 3 [file 44318_2024_172_MOESM5_ESM.zip › Figure 3/3F/Fig.3F_Non Wound.tif]

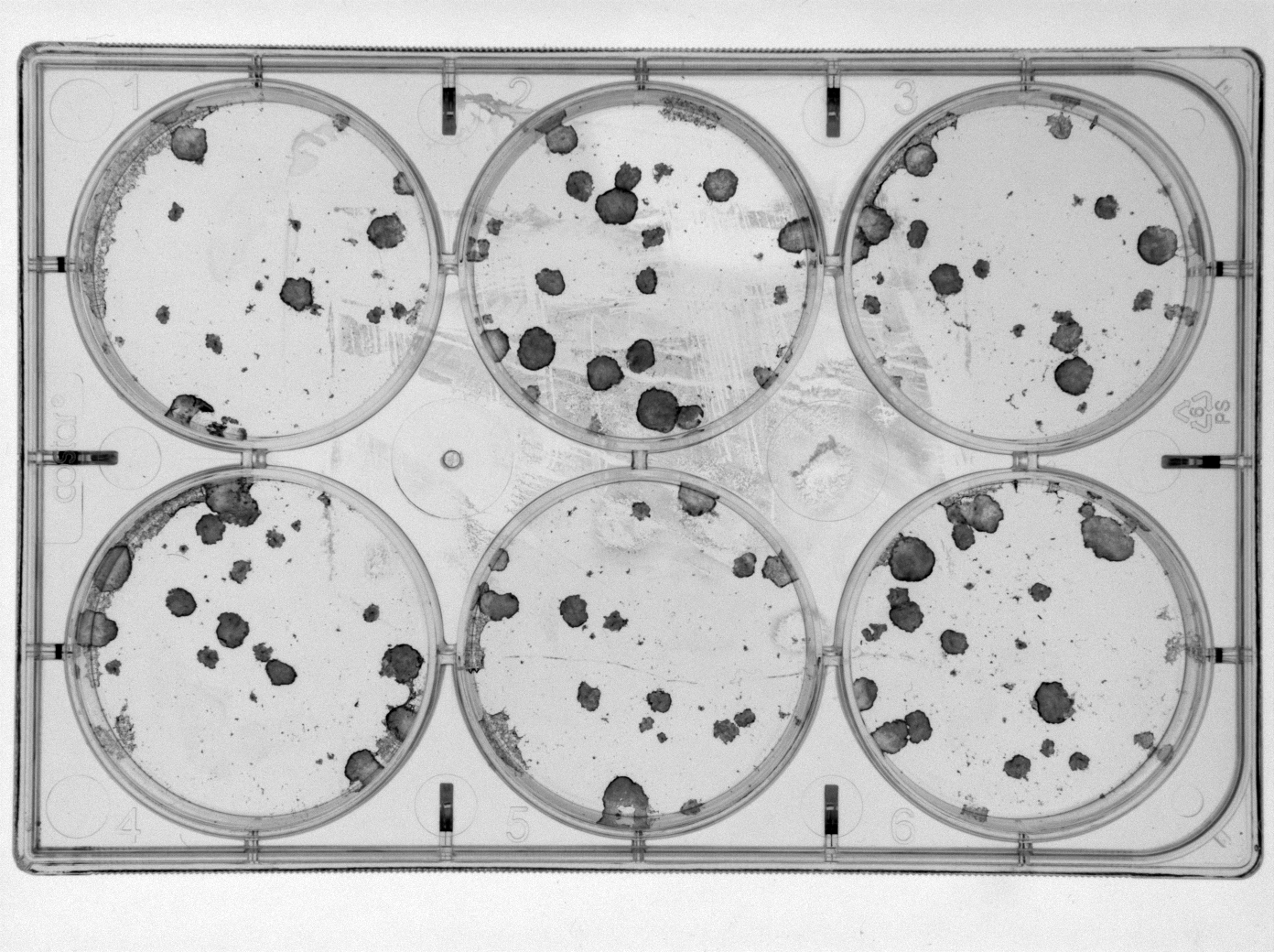

Supplement: Supplementary file 5 — Source data Fig. 3 [file 44318_2024_172_MOESM5_ESM.zip › Figure 3/3I/Fig.3I_hSNHG7-siSNHG7.tif]

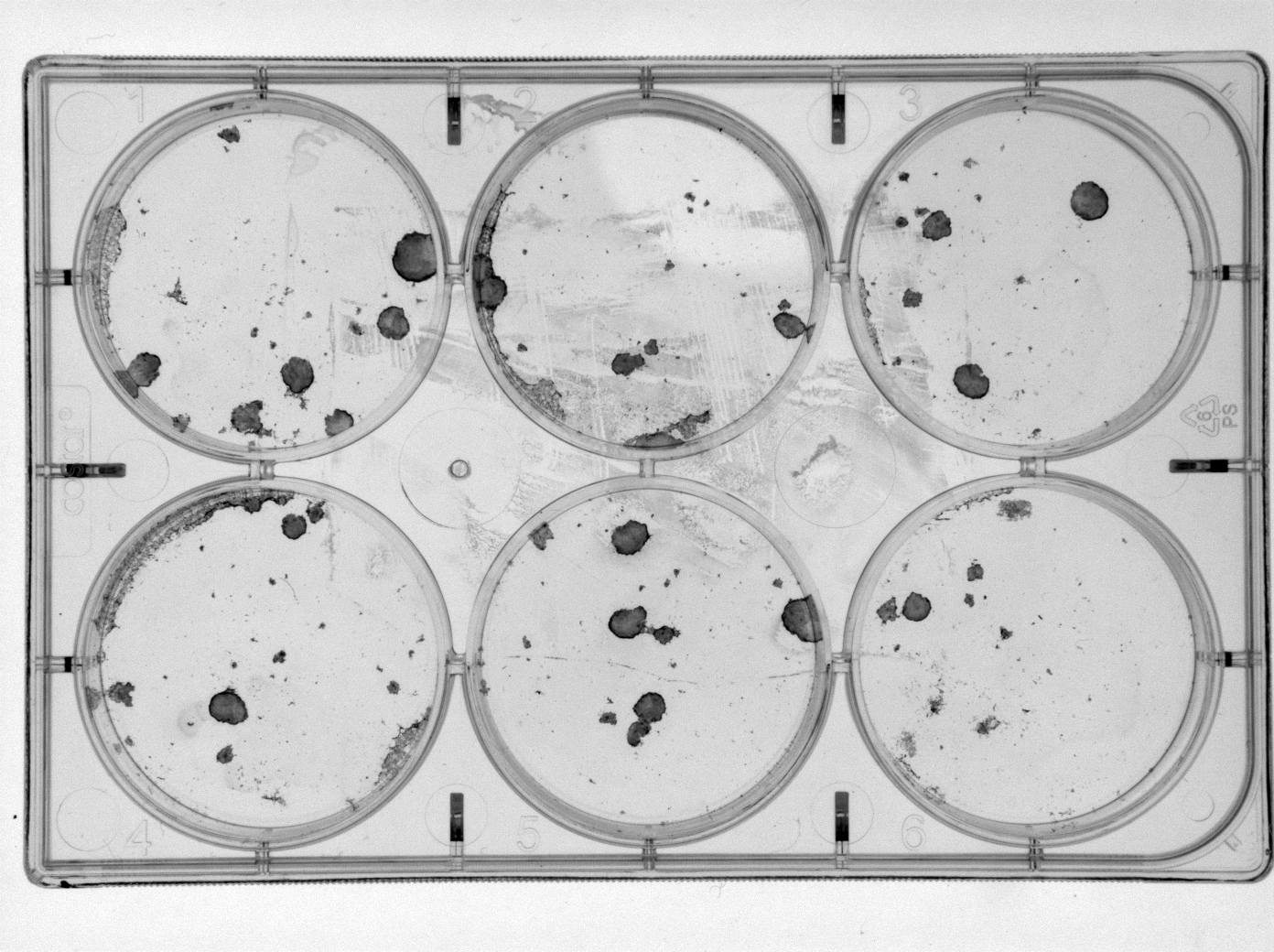

Supplement: Supplementary file 5 — Source data Fig. 3 [file 44318_2024_172_MOESM5_ESM.zip › Figure 3/3I/Fig.3I_GFP only-siSNHG7.tif]

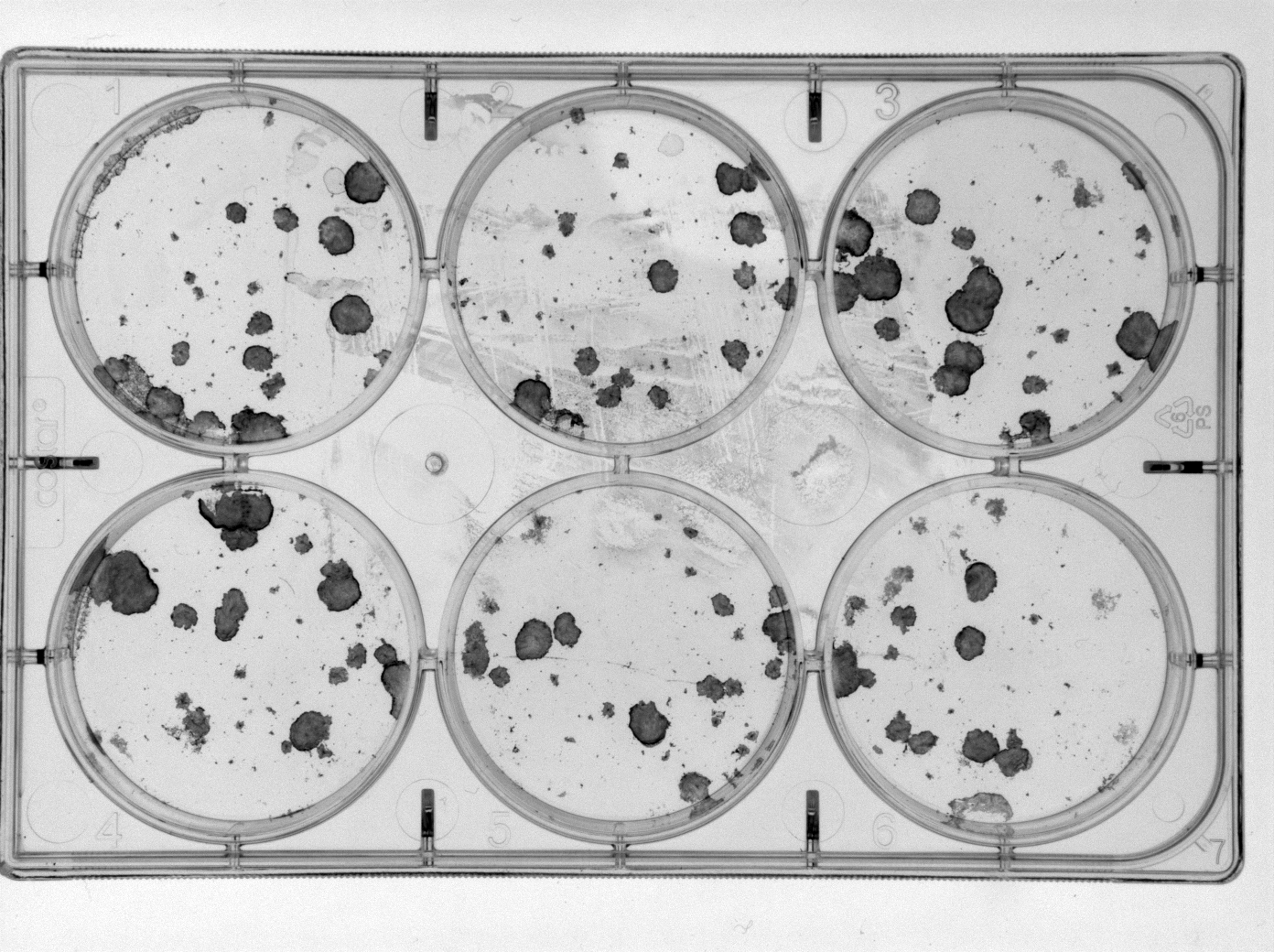

Supplement: Supplementary file 5 — Source data Fig. 3 [file 44318_2024_172_MOESM5_ESM.zip › Figure 3/3I/Fig.3I_GFP only-siScramble.tif]

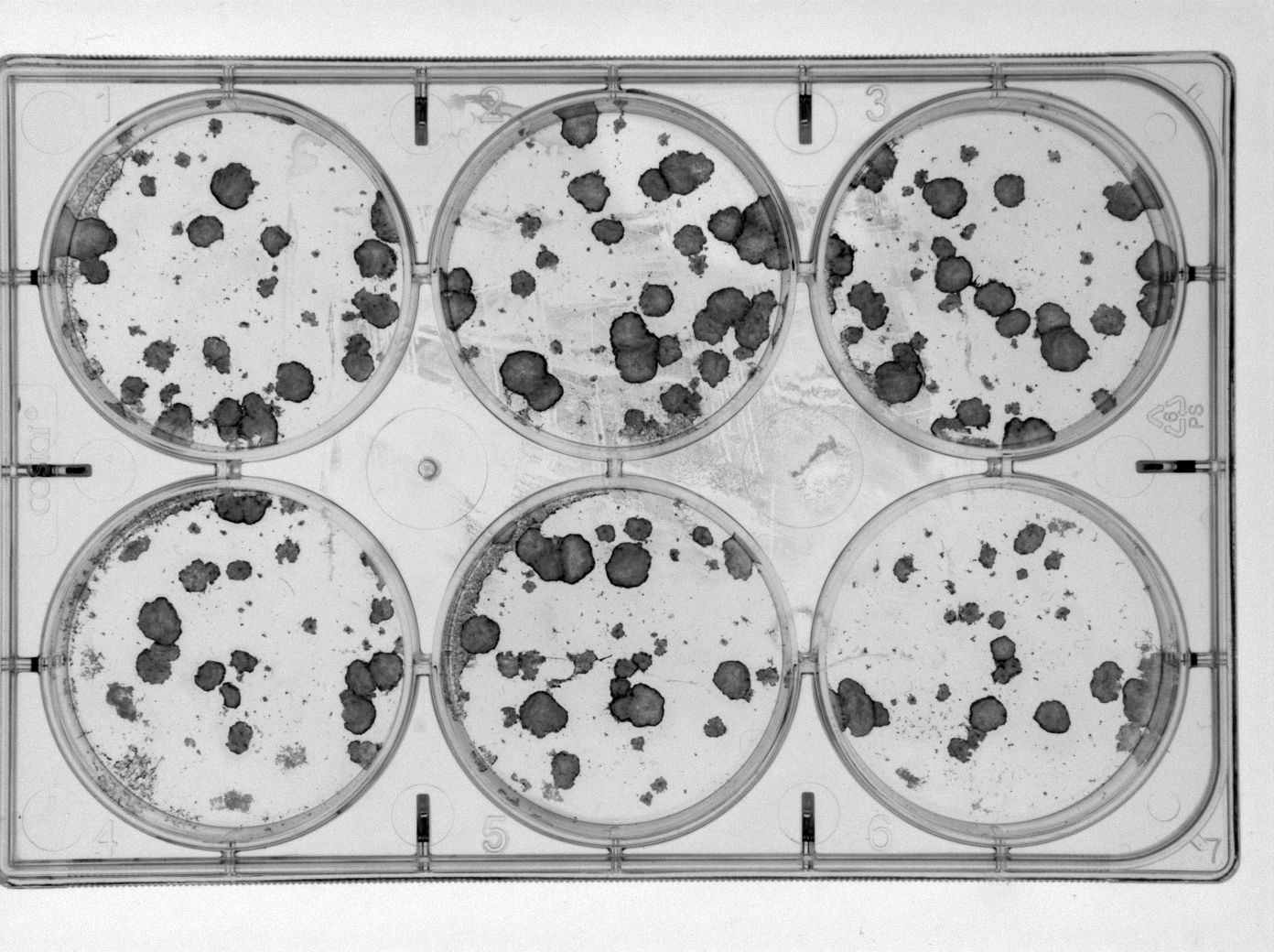

Supplement: Supplementary file 5 — Source data Fig. 3 [file 44318_2024_172_MOESM5_ESM.zip › Figure 3/3I/Fig.3I_hSNHG7-siScramble.tif]

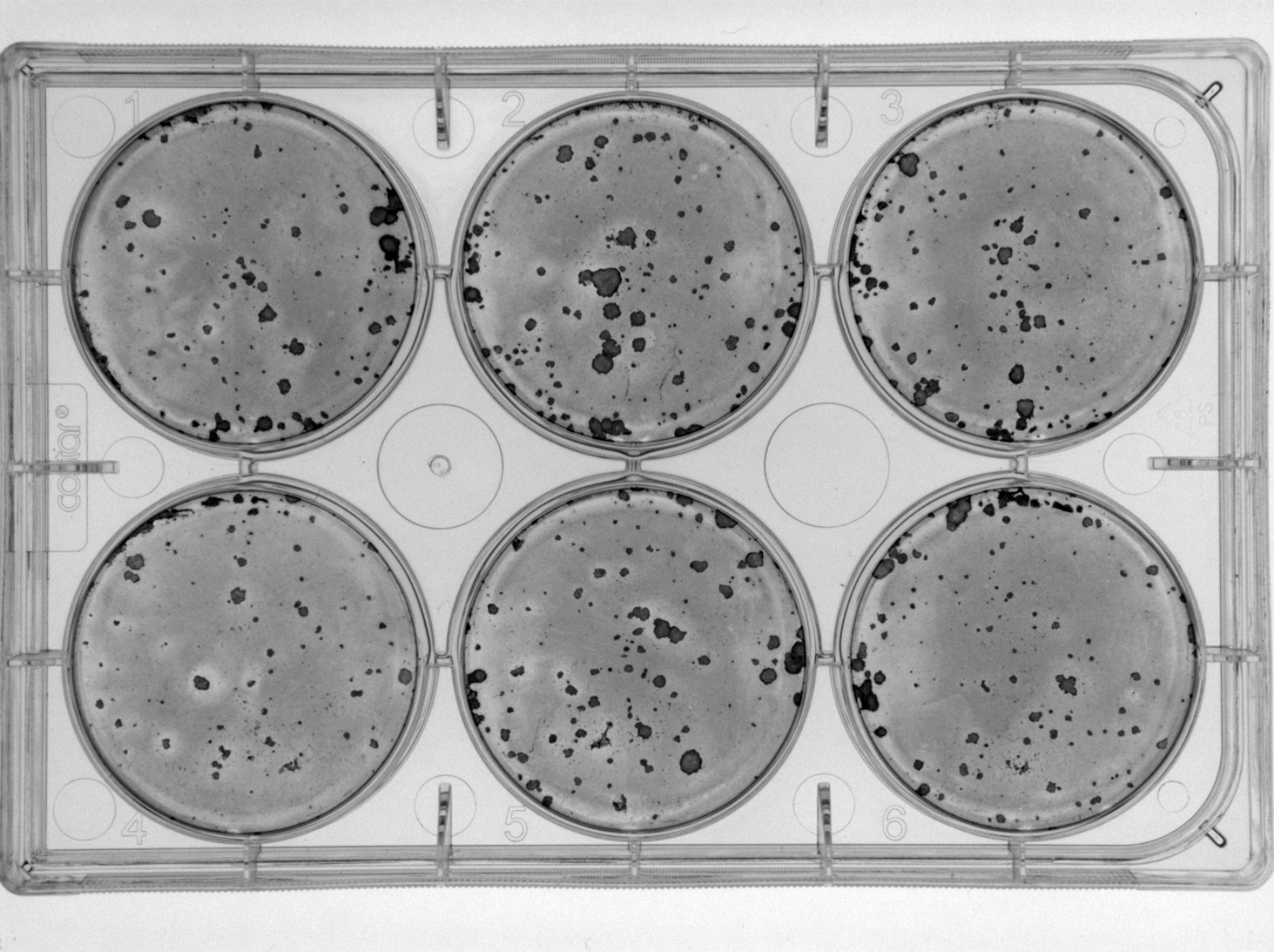

Supplement: Supplementary file 6 — Source data Fig. 4 [file 44318_2024_172_MOESM6_ESM.zip › Figure 4/4B/Fig.4B_hSNHG7.tif]

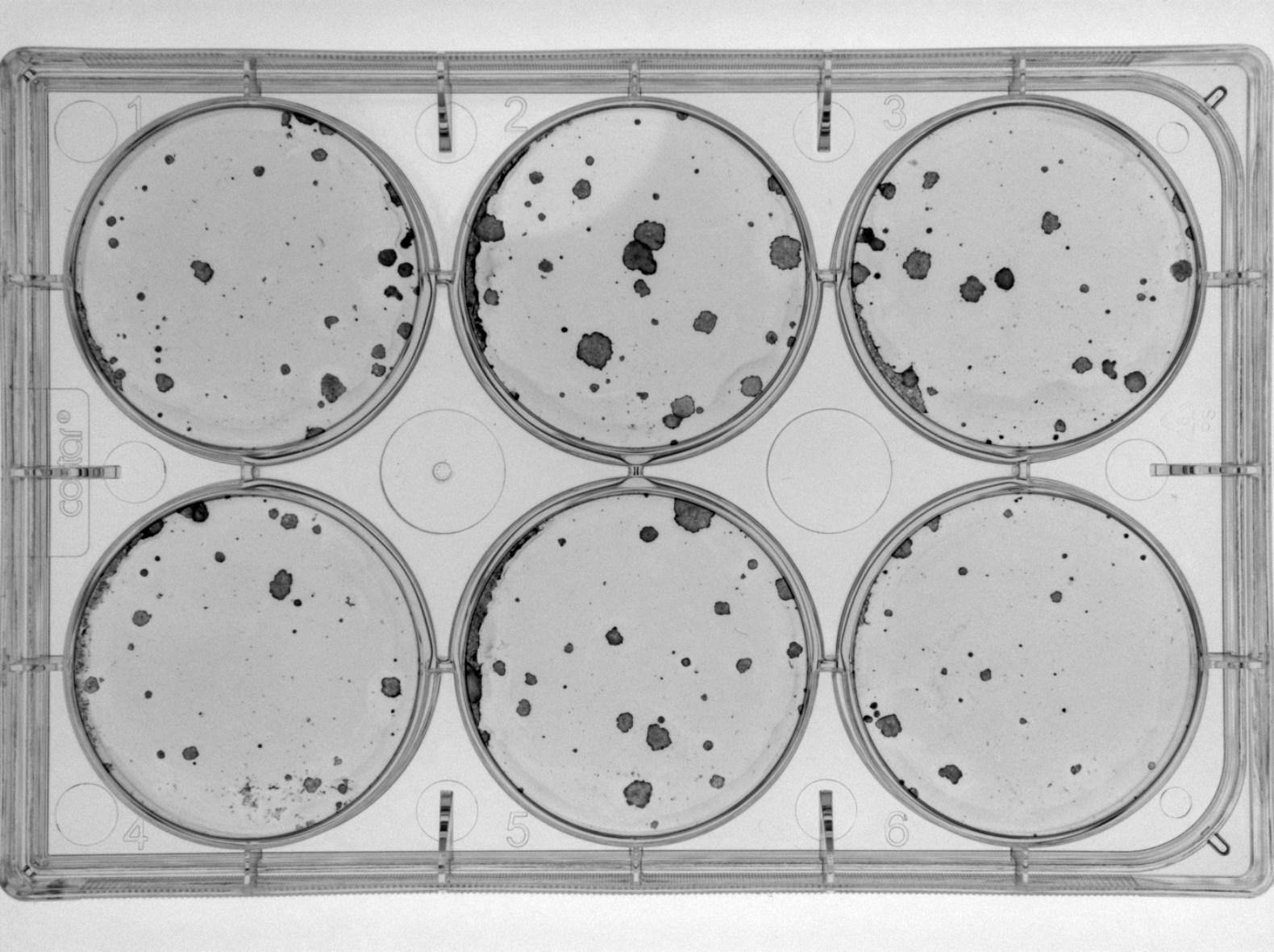

Supplement: Supplementary file 6 — Source data Fig. 4 [file 44318_2024_172_MOESM6_ESM.zip › Figure 4/4B/Fig.4B_siSNHG7.tif]

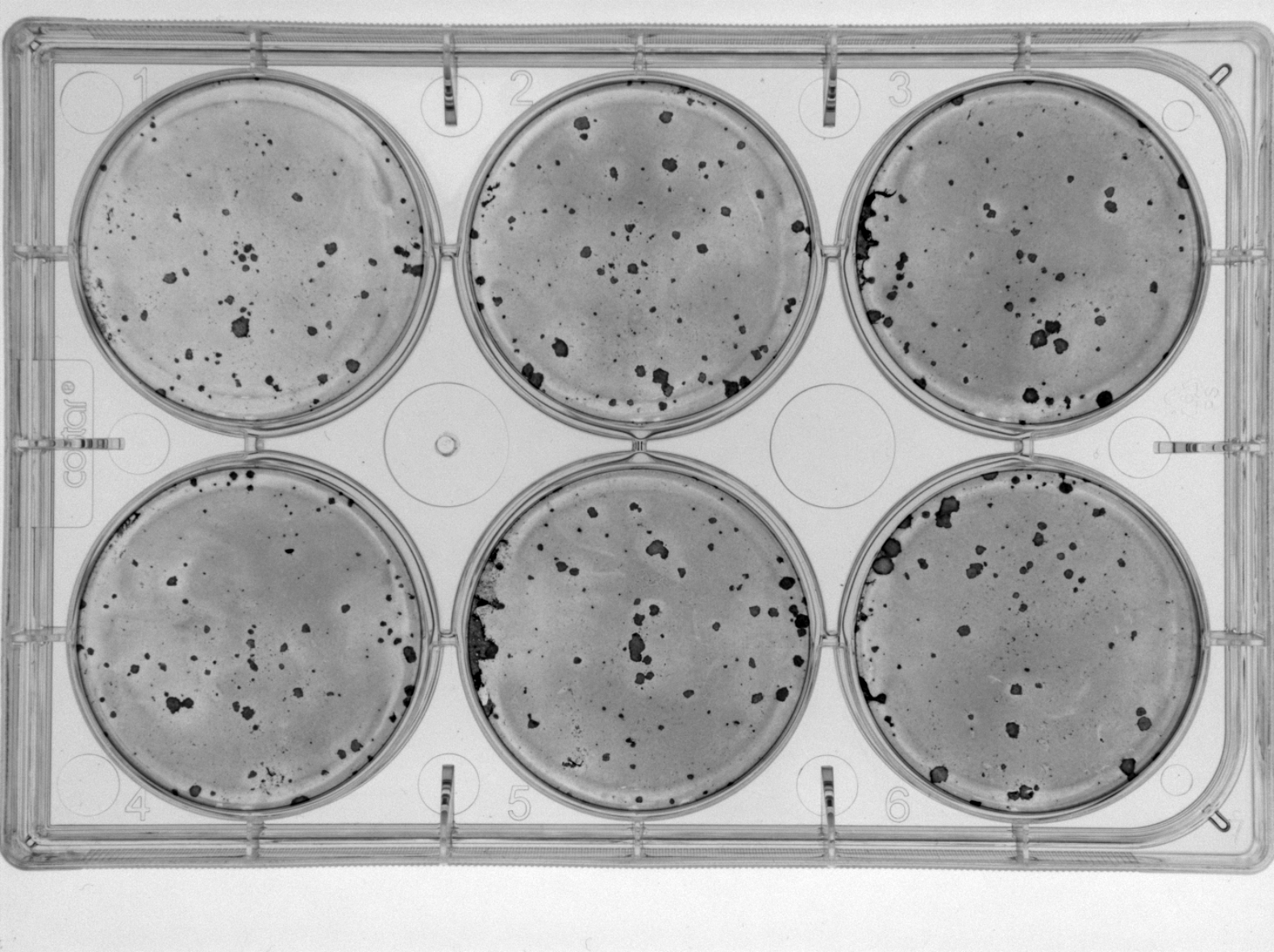

Supplement: Supplementary file 6 — Source data Fig. 4 [file 44318_2024_172_MOESM6_ESM.zip › Figure 4/4B/Fig.4B_GFP only.tif]

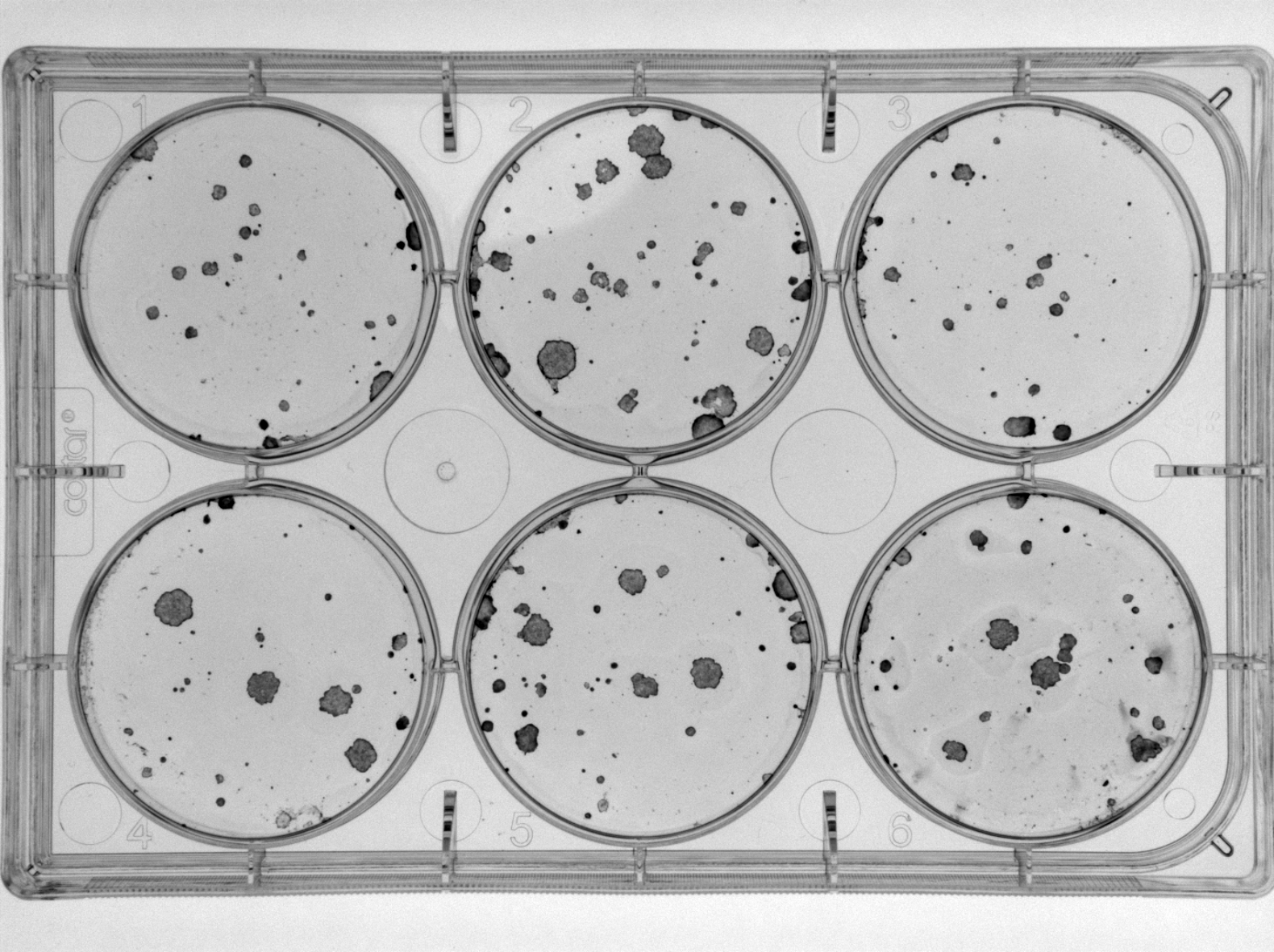

Supplement: Supplementary file 6 — Source data Fig. 4 [file 44318_2024_172_MOESM6_ESM.zip › Figure 4/4B/Fig.4B_siScramble.tif]

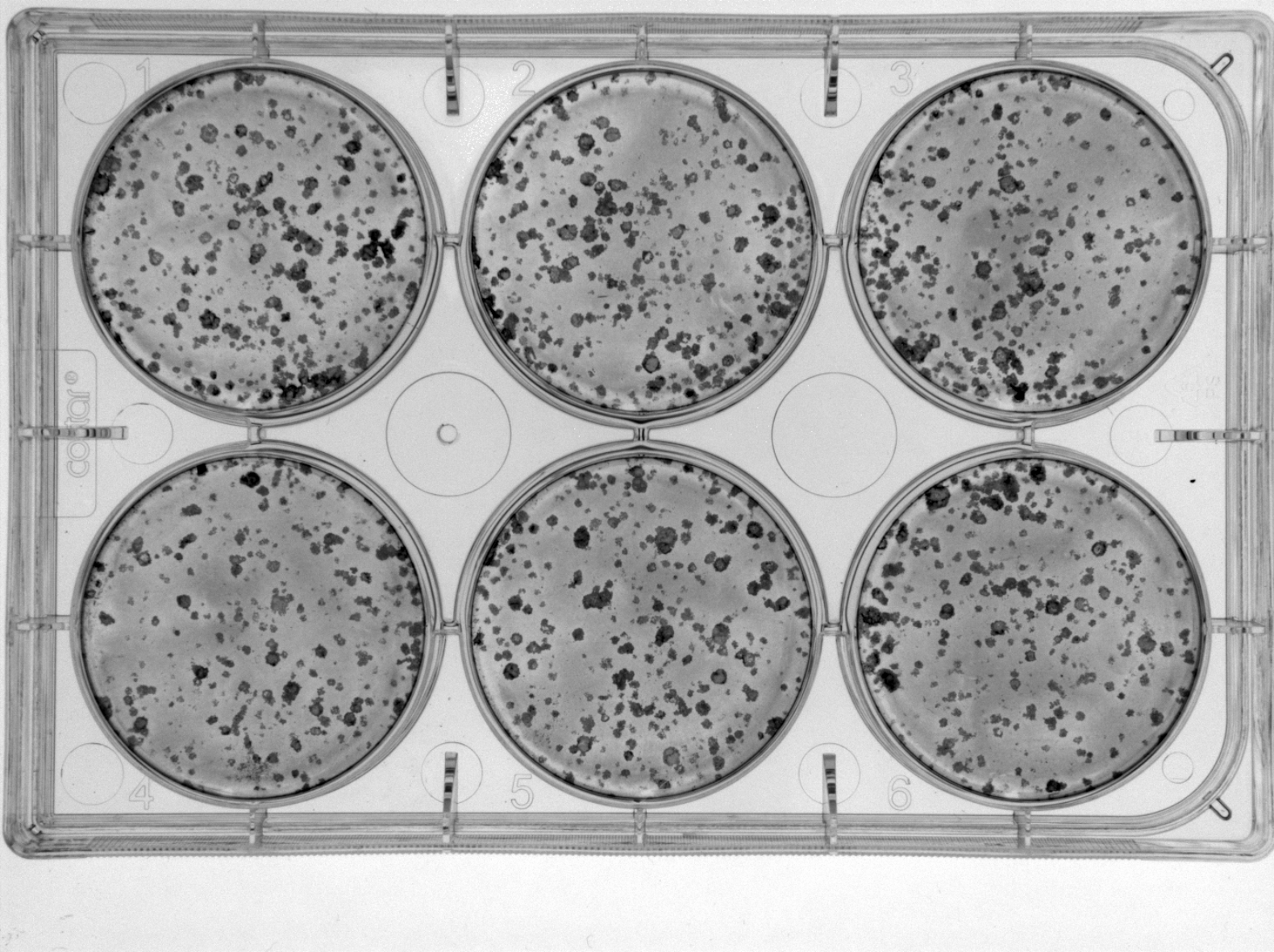

Supplement: Supplementary file 6 — Source data Fig. 4 [file 44318_2024_172_MOESM6_ESM.zip › Figure 4/4C/Fig.4C_GFP only.tif]

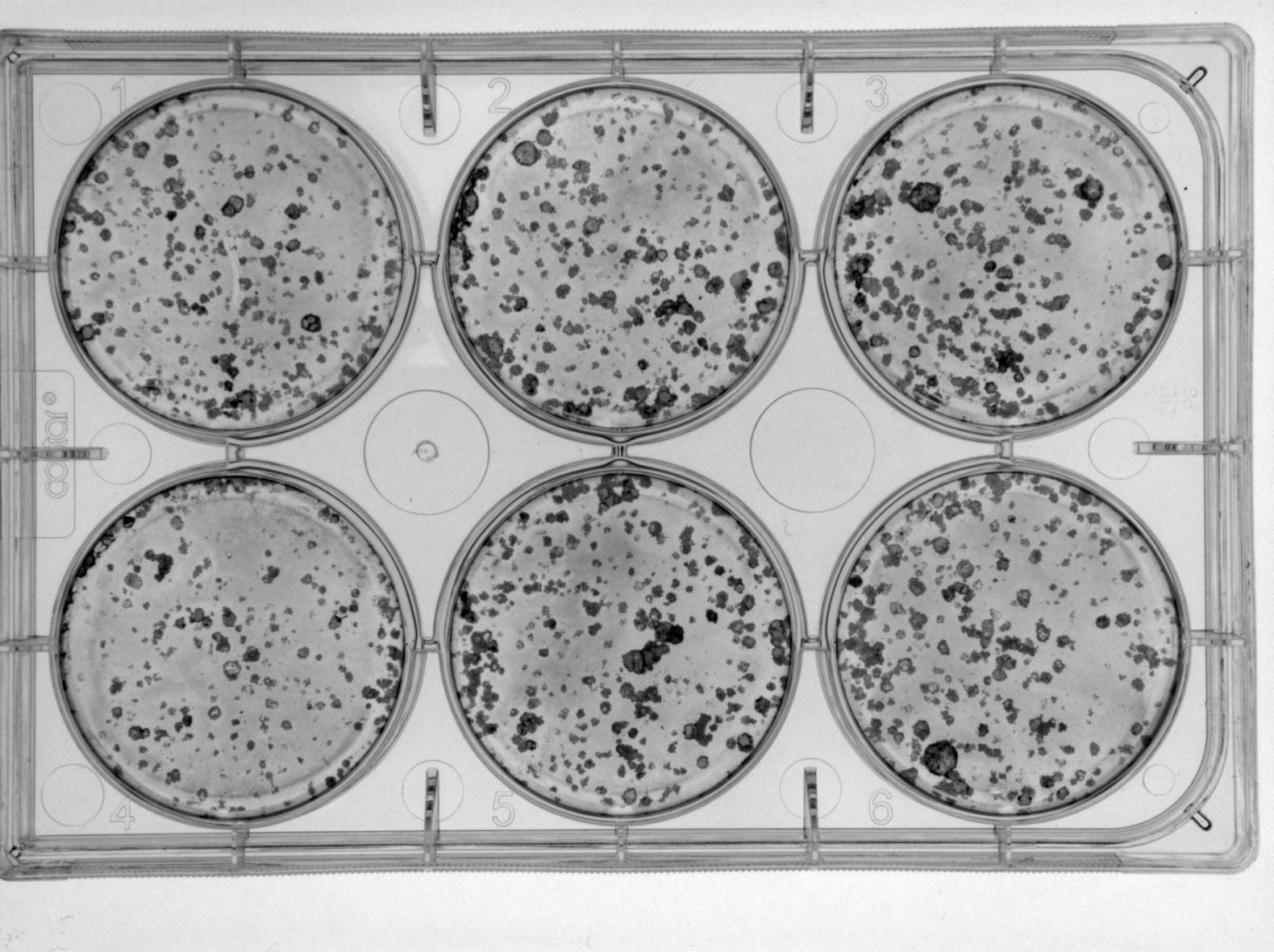

Supplement: Supplementary file 6 — Source data Fig. 4 [file 44318_2024_172_MOESM6_ESM.zip › Figure 4/4C/Fig.4C_hSNHG7.tif]

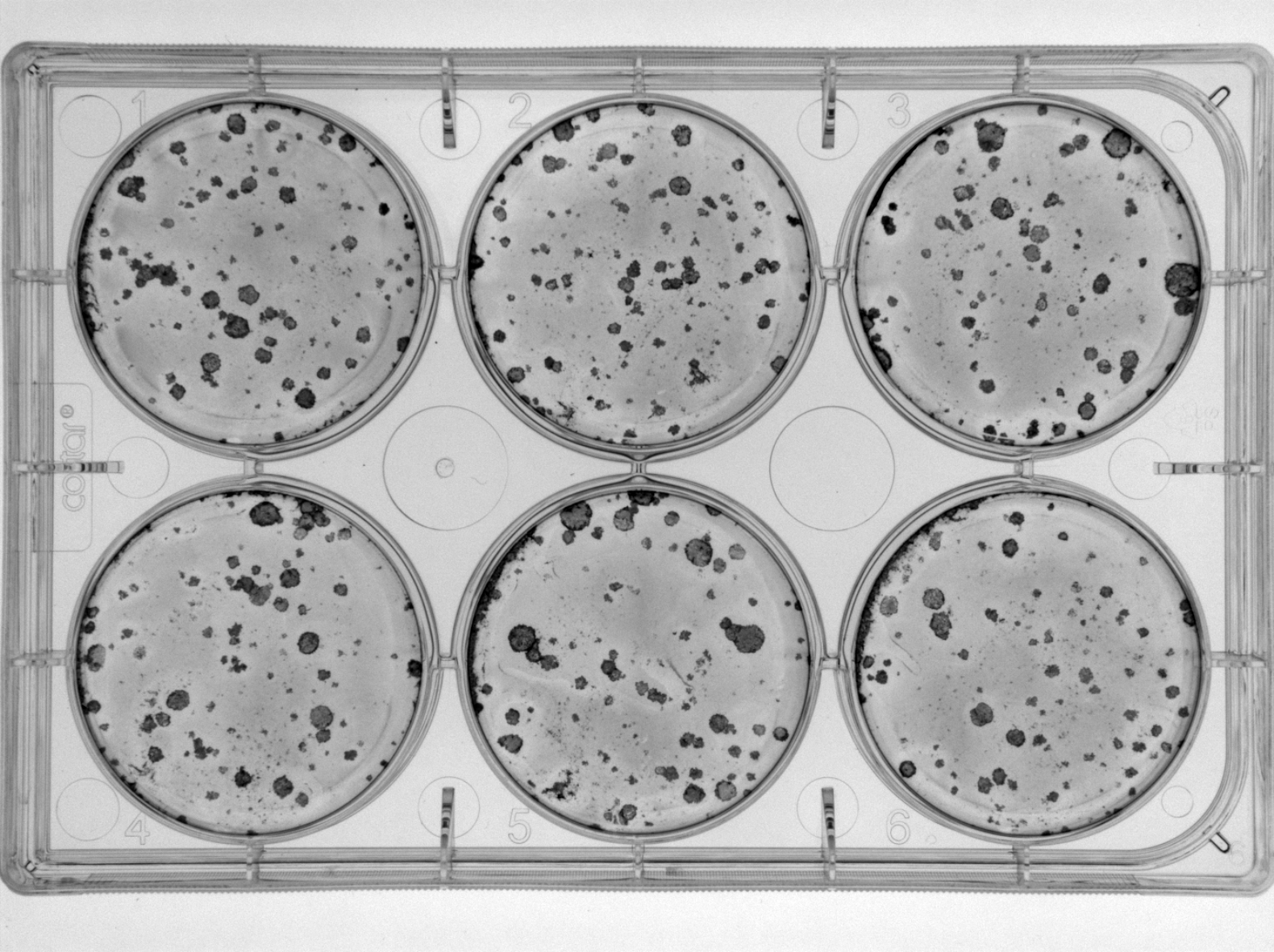

Supplement: Supplementary file 6 — Source data Fig. 4 [file 44318_2024_172_MOESM6_ESM.zip › Figure 4/4C/Fig.4C_siScramble.tif]

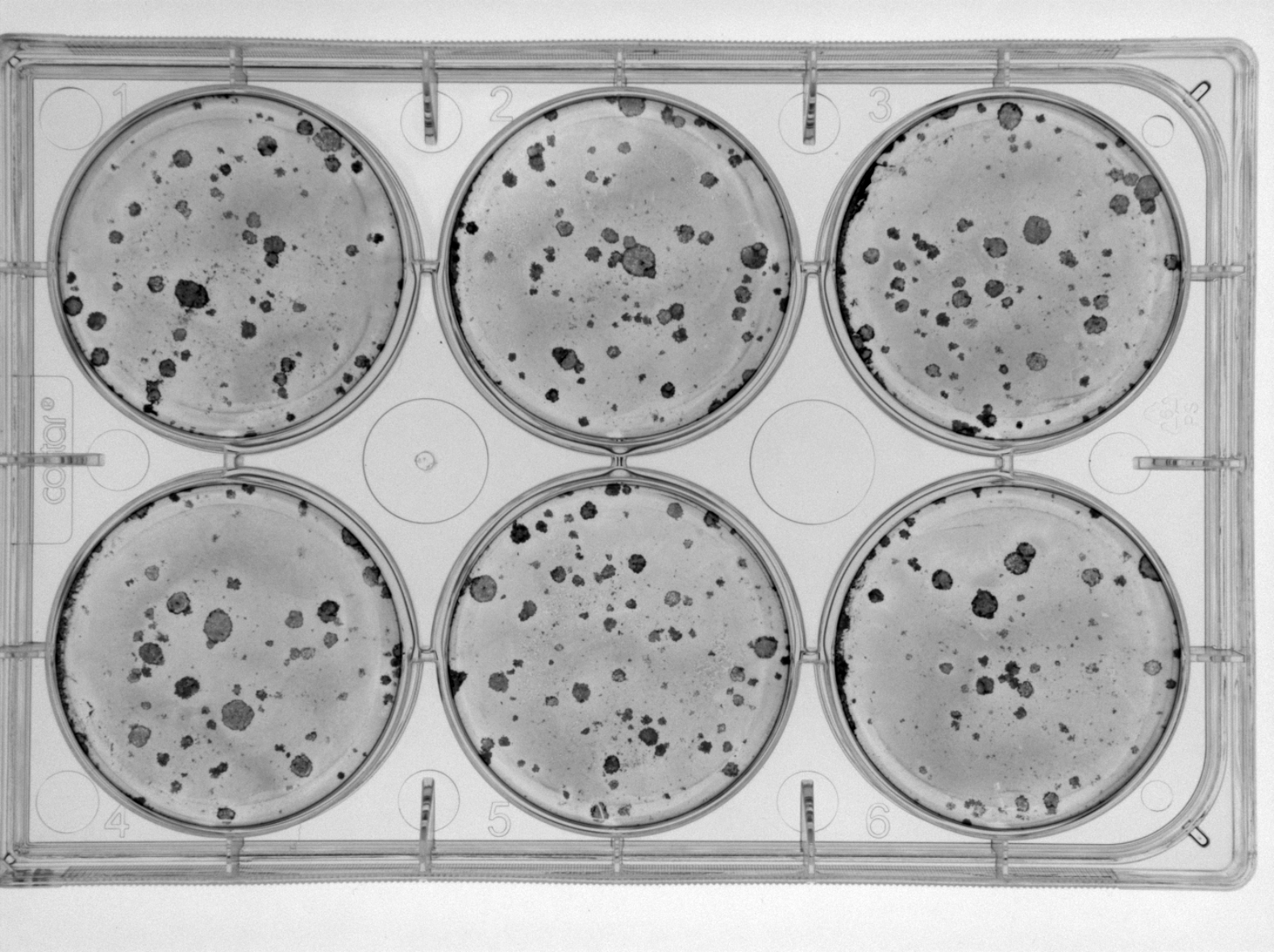

Supplement: Supplementary file 6 — Source data Fig. 4 [file 44318_2024_172_MOESM6_ESM.zip › Figure 4/4C/Fig.4C_siSnhg7.tif]

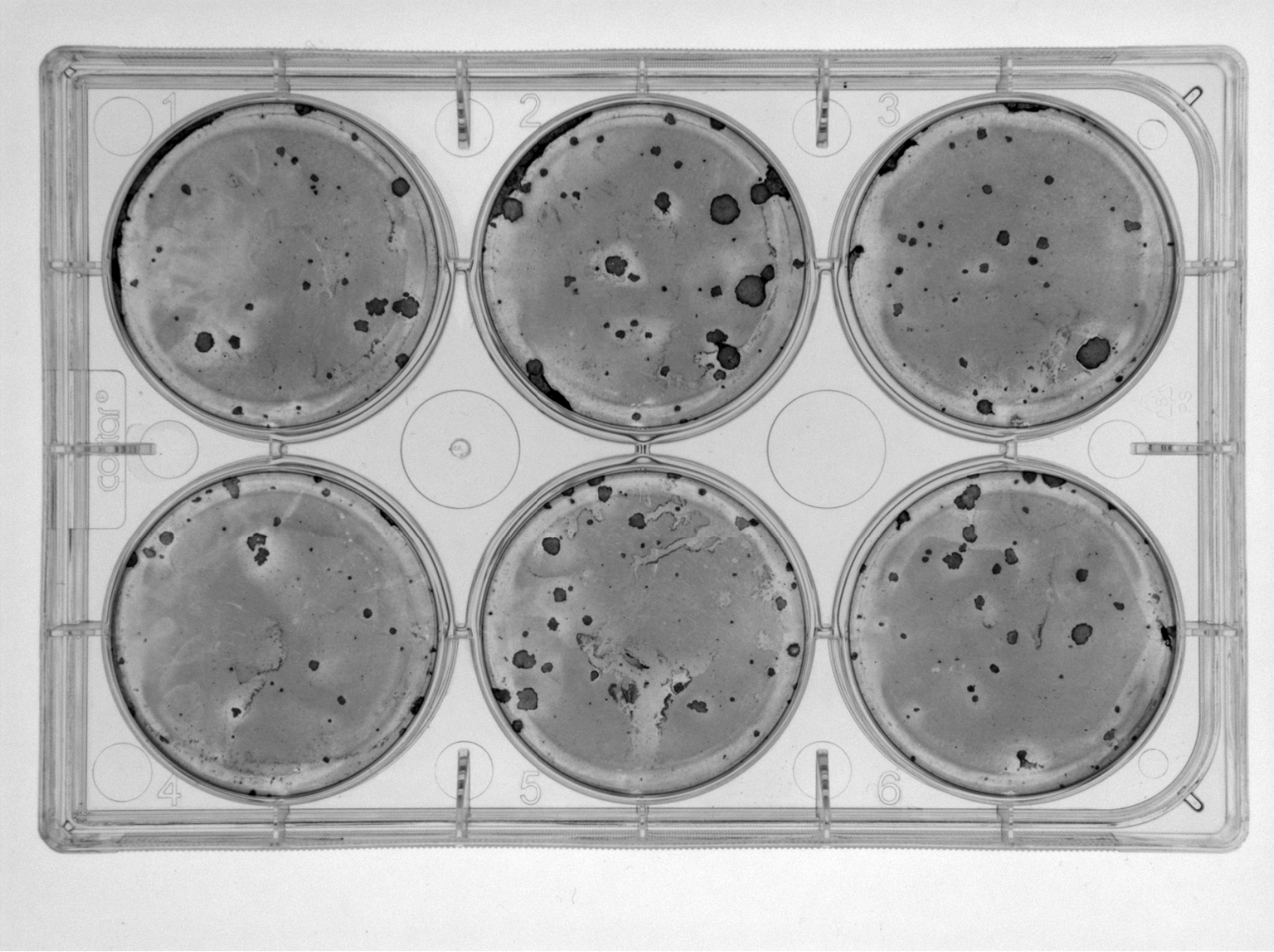

Supplement: Supplementary file 8 — Source data Fig. 6 [file 44318_2024_172_MOESM8_ESM.zip › Figure 6/6B/Fig.6B_hSNHG7mut34.tif]

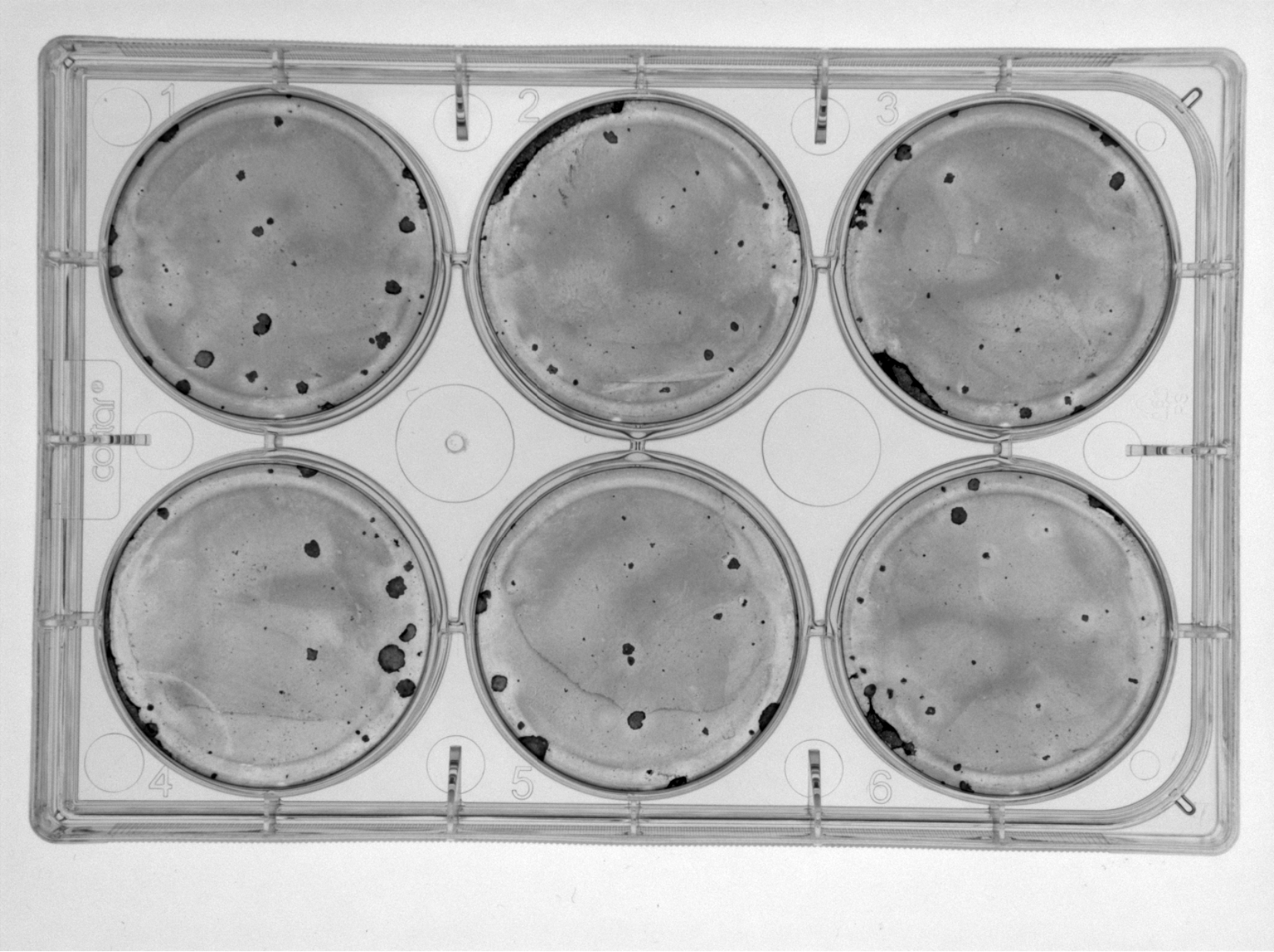

Supplement: Supplementary file 8 — Source data Fig. 6 [file 44318_2024_172_MOESM8_ESM.zip › Figure 6/6B/Fig6B_GFP only.tif]

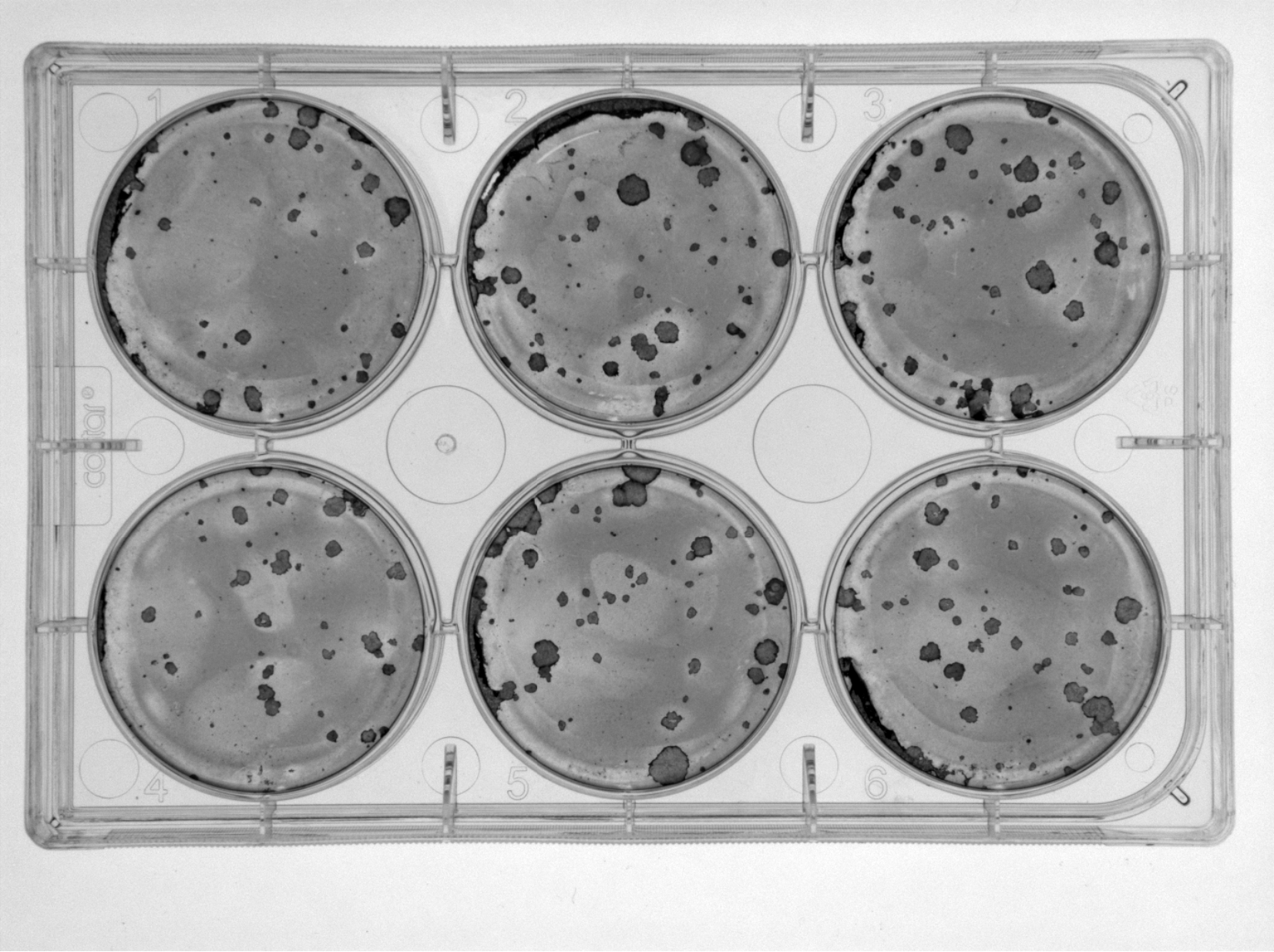

Supplement: Supplementary file 8 — Source data Fig. 6 [file 44318_2024_172_MOESM8_ESM.zip › Figure 6/6B/Fig.6B_hSNHG7mutCTR.tif]

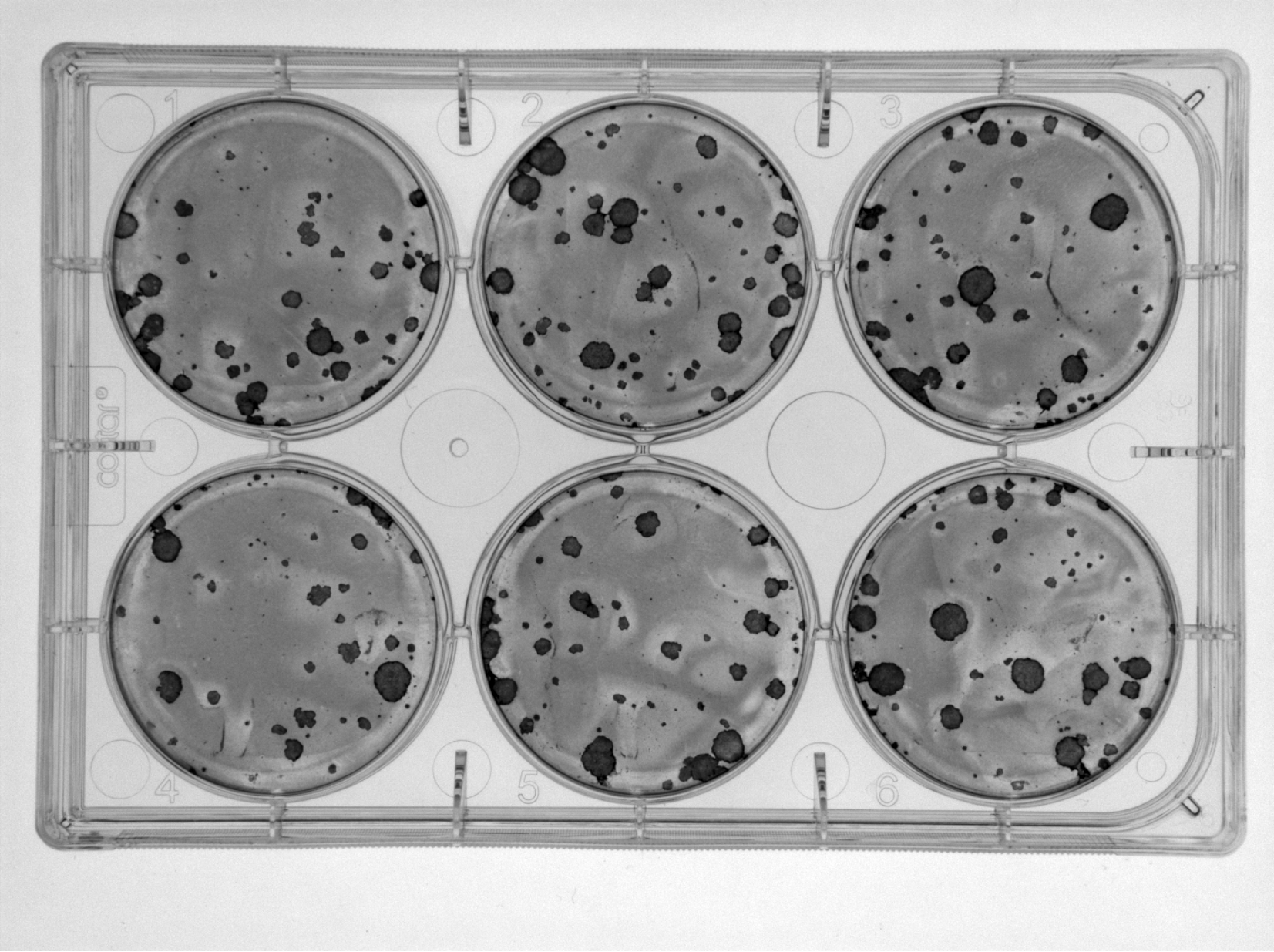

Supplement: Supplementary file 8 — Source data Fig. 6 [file 44318_2024_172_MOESM8_ESM.zip › Figure 6/6B/Fig.6B_hSNHG7mut193.tif]

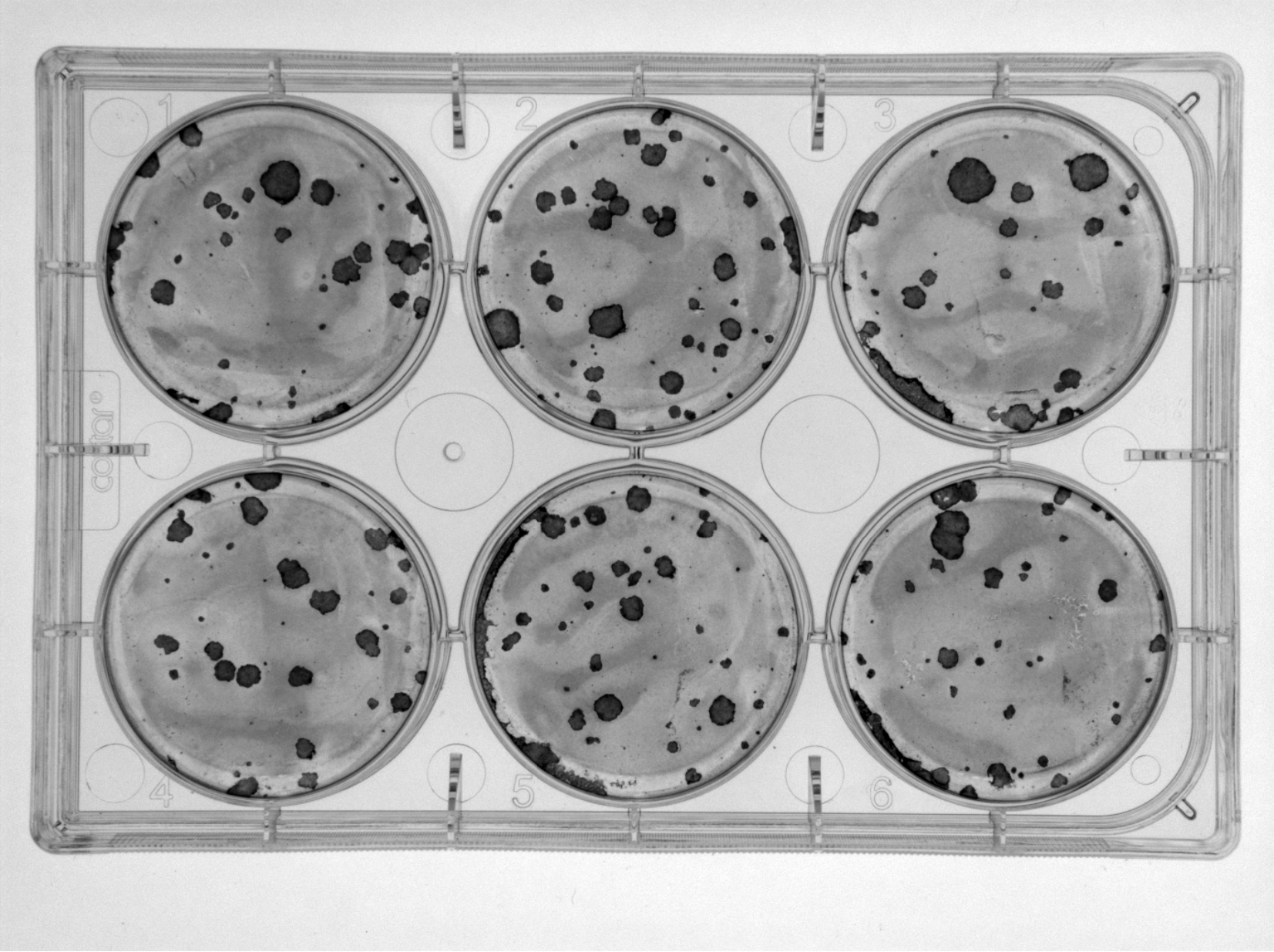

Supplement: Supplementary file 8 — Source data Fig. 6 [file 44318_2024_172_MOESM8_ESM.zip › Figure 6/6B/Fig.6B_hSNHG7.tif]

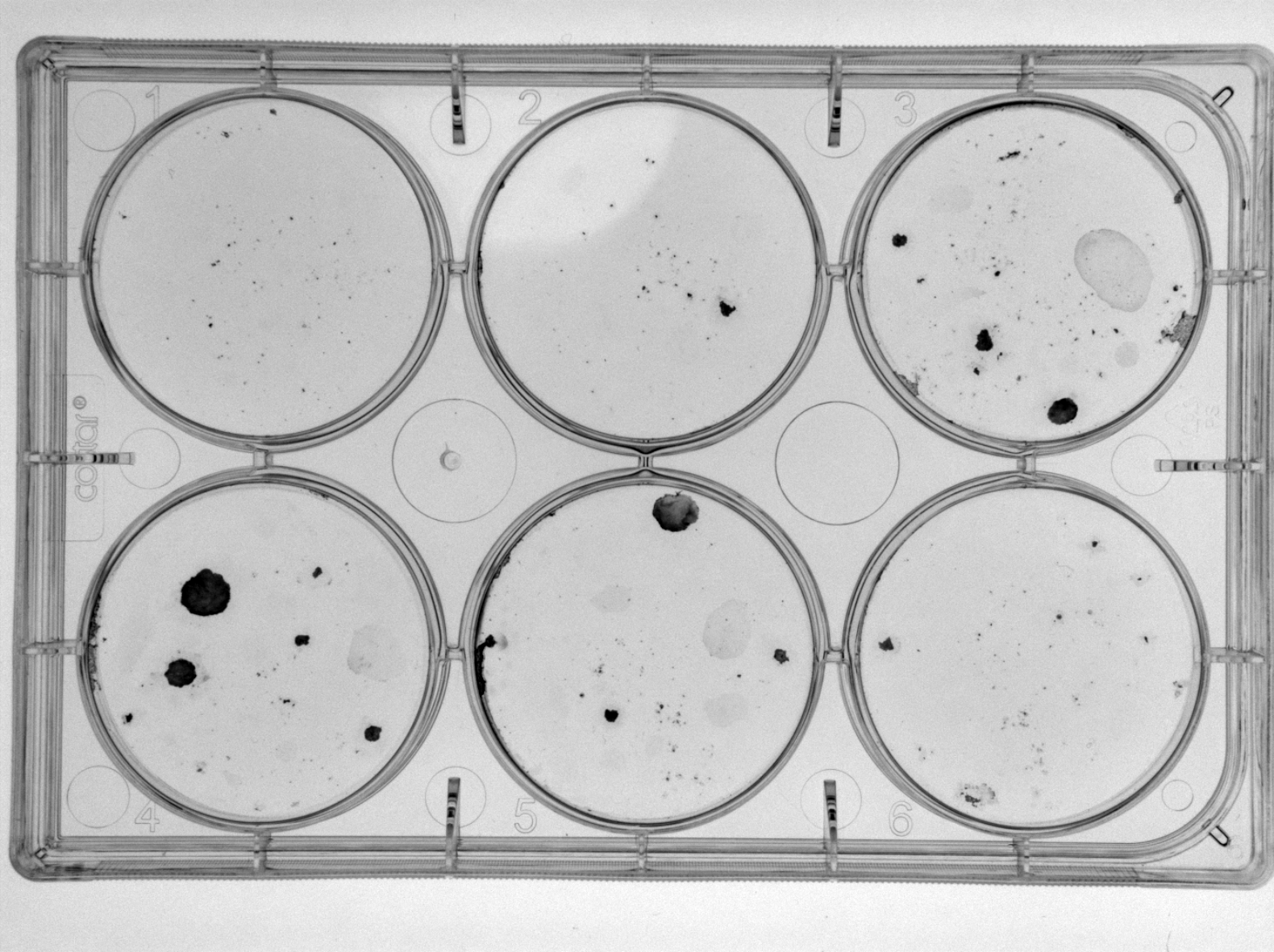

Supplement: Supplementary file 8 — Source data Fig. 6 [file 44318_2024_172_MOESM8_ESM.zip › Figure 6/6E/Fig.6E_GFP only siSNHG7.tif]

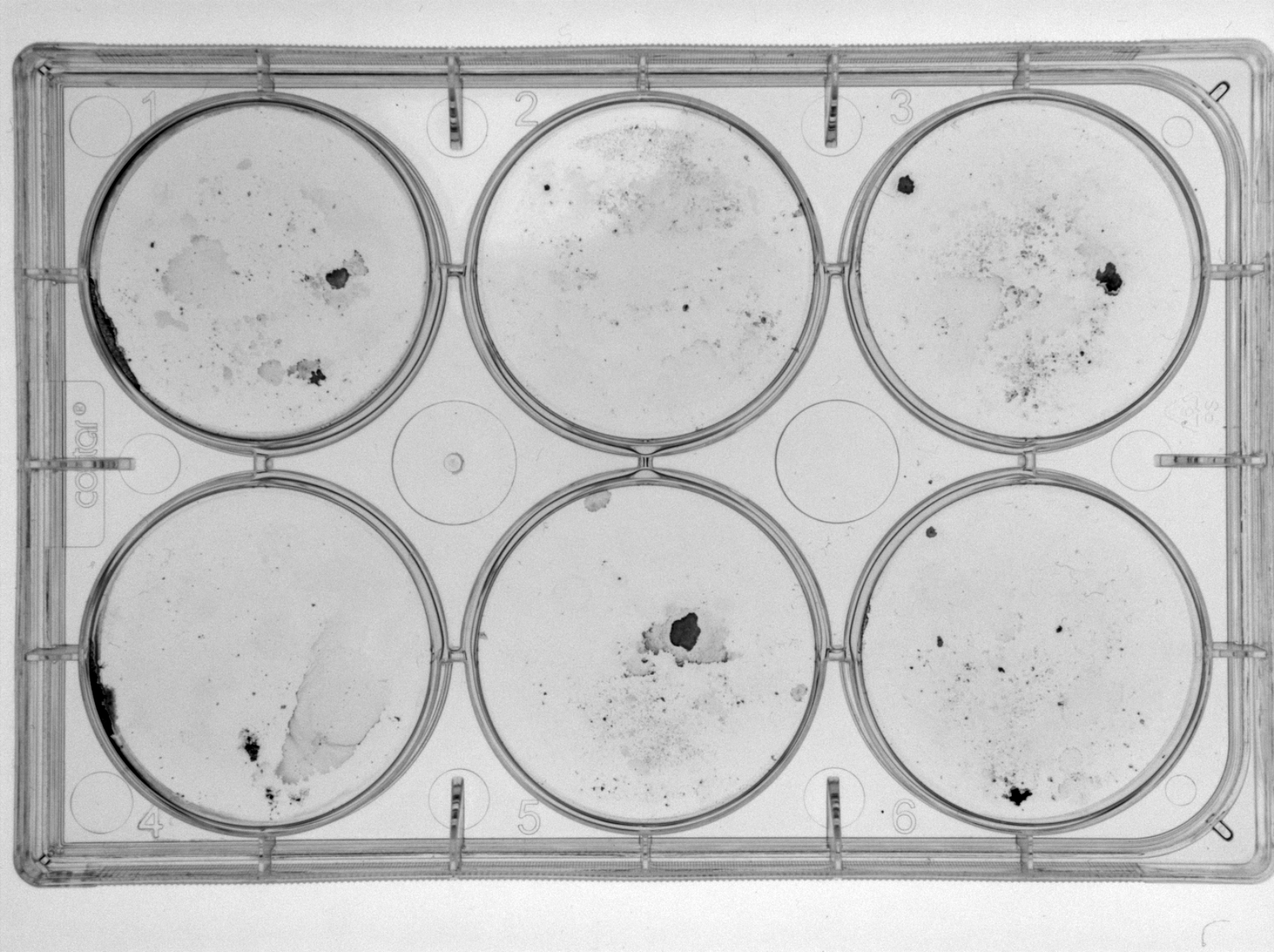

Supplement: Supplementary file 8 — Source data Fig. 6 [file 44318_2024_172_MOESM8_ESM.zip › Figure 6/6E/Fig.6E_hSNHG7mut34 siSNHG7.tif]

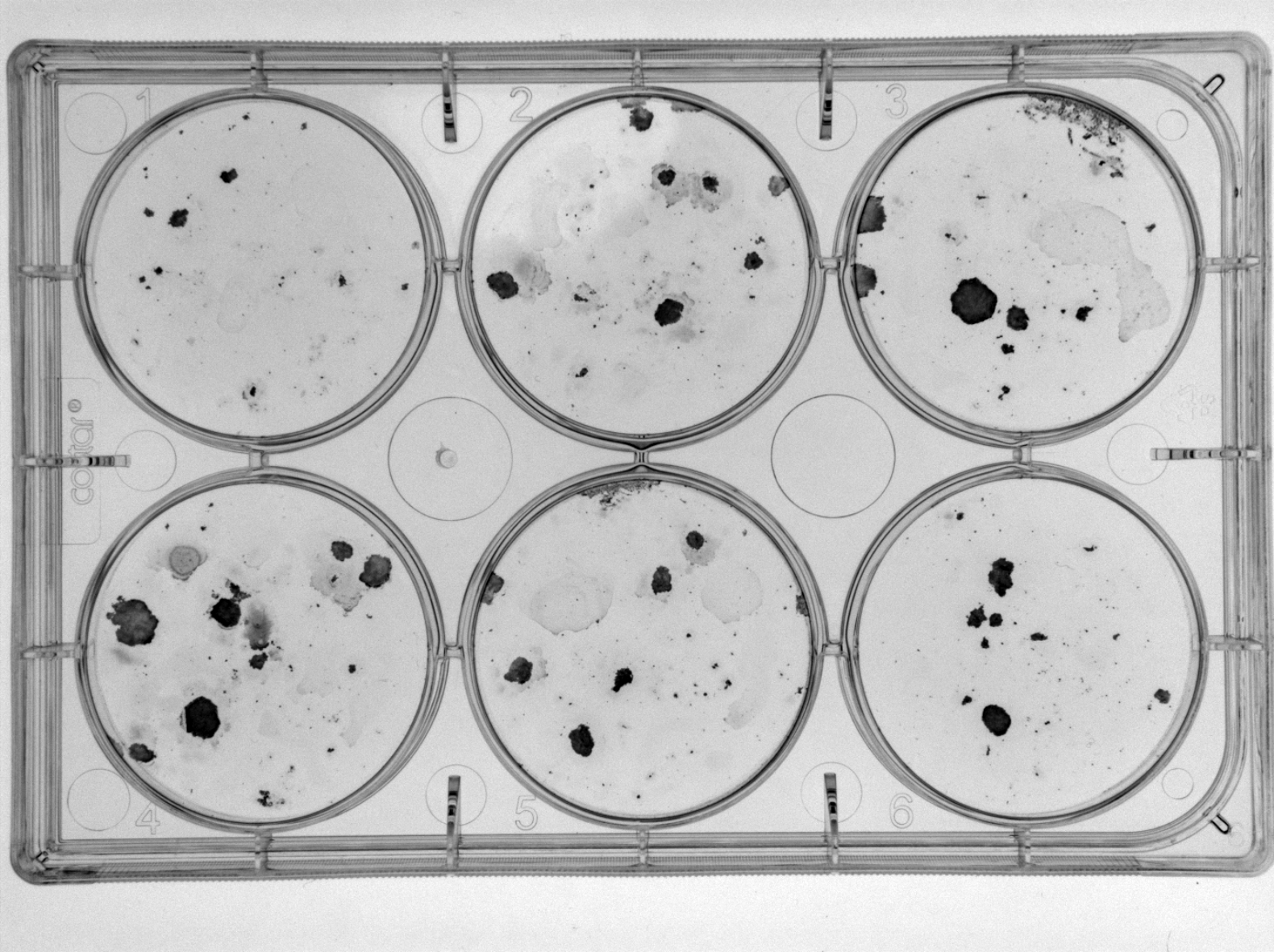

Supplement: Supplementary file 8 — Source data Fig. 6 [file 44318_2024_172_MOESM8_ESM.zip › Figure 6/6E/Fig.6E_GFP only siScramble.tif]
