# Supplementary material for: Neutral evolution of snoRNA Host Gene long non-coding RNA affects cell fate control
Source: EMBO J. 2024 Jul 25;43(18):4049–67. doi: 10.1038/s44318-024-00172-8 (PMC11405852; doi:10.1038/s44318-024-00172-8)
Supplement: Supplementary file 6 — Source data Fig. 4 [file 44318_2024_172_MOESM6_ESM.zip › Figure 4/4A/Fig4A_Atri SNHG7 sequences.docx]

>Aotus trivirgatus SNHG7 (transcript 1)

CTCTGCGCGCGCCGGCGGCTGCCATGGCGGGACGTCTGCTCACTGGAAACACACGTGCGAGGGGTCCTGGGGTGCTGGGAAGGAGGTGACTTAGCCTGTGATGGACTTCCAGTGTGAGCAGTGGCCAGAGTGACGAGGCCAACCGGCCCCAGTCCGAATCAAGATGCAGAGGCCAGGATGTGGGCGCAGCCCCGTGCCAAGAGGCGGGCTGGCATAGGACCTCCGGCACCCAGGCTGTTTGCGGCCTCAGAGCCCAGCTTTCCGCACGCCCACCTGCCCCCAGGGCCACGGTTGCAGCTCCTGCTCTGCCTGCATTCCAGGGATGGGCAGGCTGGCATCGGGACGCCCACCGCCTCTGCCTGGGTAGTGCTGTGTGTTCCAGCCGGCCAGGGCAGCTGCCAGGACCACCCCTCCATTTGAGTATCCCGGTTCTTAAGTTCTGCCATTGTGGTGTTCTGCTGGAAAAAGAACCATTTGGCTGTGTCTGAACTGCCTGGAACCCAAGATCCCGAGTTATTTTTTACTGTATTTGAGTCATCTTGTGTTTGTTGTTTTTACCCCAAGGGGAAAATCTAGATGGAAAACATTTATTTTAAAATACAGGATGAAGGGAATTAAAAGATTTAATGCACATTTCTTCAAGGATAGTATTTCTGTATTGGCAAAATTTGAGAATAAATGGGTCTGGAACGAAAAAAAAAAAAAAA

>Aotus trivirgatus SNHG7 (transcript 2)

CGCGTGAGCCGCGGGATGGGGGCCCGGGCCCGGGAGGAGGCGCCGTGCTGTGTCCCTCCCCGCGCGGTTTCCAGCCGGGAAGCTTCGGGAAGCCTGGTGAGGGCCGAGTGCGCGAATTCGGACCTAAGCGGAAAGCGCCCCGYCCACCCGCGCCCCTTCCGCCCCTGCTGCTGCGTCCCCGAGTCGCGGAGGCTCTGGGGACGTCGCCTCCTGTGTCGGCATCTTCGAGAAATGGATTTCTCGTGCCGTGTCCACGCGTCGGGTGTTTCCGTGTGACTGGCCGCTCAGCGGGAGGCTGTCCTGGCGGAAGGAGTCCGGTGACCCCCGGACTAAATACTGTTACAGGACAGGTGCGCGCCTGTCCTTGGGGGGCATCCGCCTCGTGGTCCTGGTCCCTGGACACCTGCAGGGGATCAGCCTTCCGTGGCCACTGTTGTGACTCAACTTCCCCTCCTGCAGATTAAGGAGAGAGACGTCTGCTCACTGGAAACACACGTGCGAGGGGTCCTGGGGTGCTGGGAAGGAGGTGACTTAGCCTGTGATGGACTTCCAGTGTGAGCAGTGGCCAGAGTGACGAGGCCAACCGGCCCCAGTCCGAATCAAGATGCAGAGGCCAGGATGTGGGCGCAGCCCCGTGCCAAGAGGCGGGCTGGCATAGGACCTCCGGCACCCAGGCTGTTTGCGGCCTCAGAGCCCAGCTTTCCGCACGCCCACCTGCCCCCAGGGCCACGGTTGCAGCTCCTGCTCTGCCTGCATTCCAGGGATGGGCAGGCTGGCATCGGGACGCCCACCGCCTCTGCCTGGGTAGTGCTGTGTGTTCCAGCCGGCCAGGGCAGCTGCCAGGACCACCCCTCCATTTGAGTATCCCGGTTCTTAAGTTCTGCCATTGTGGTGTTCTGCTGGAAAAAGAACCATTTGGCTGTGTCTGAACTGCCTGGAACCCAAGATCCCGAGTTATTTTTTACTGTATTTGAGTCATCTTGTGTTTGTTGTTTTTACCCCAAGGGGAAAATCTAGATGGAAAACATTTATTTTAAAATACAGGATGAAGGGAATTAAAAGATTTAATGCACATTTCTTCAAGGATAGTATTTCTGTATTGGCAAAATTTGAGAATAAATGGGTCTGGAACGAAAAAAAAAAAAAAA
